# Synthesis of MeON-Glycoside Derivatives of Oleanolic Acid by Neoglycosylation and Evaluation of Their Cytotoxicity against Selected Cancer Cell Lines

**DOI:** 10.3390/molecules26030772

**Published:** 2021-02-02

**Authors:** Zhichao Du, Guolong Li, Xiaoyang Zhou, Jian Zhang

**Affiliations:** 1 State Key Laboratory of Natural Medicines, China Pharmaceutical University, Nanjing 210009, Jiangsu, China; duzhichao1987@163.com (Z.D.); 1821020430@stu.cpu.edu.cn (X.Z.); 2 Department of Medicinal Chemistry, China Pharmaceutical University, Nanjing 211198, Jiangsu, China; 1620194533@cpu.edu.cn; 3 Zhenping Expert Workstation for Zhang Jian, Zhenping, Ankang 725699, Shaanxi, China

**Keywords:** oleanolic acid, neoglycosides, anticancer, antiproliferation, apoptosis

## Abstract

A series of C-3 and C-28 MeON-neoglycosides of oleanolic acid were designed and synthesized by neoglycosylation as potential antiproliferative agents. Their cytotoxicity was evaluated in vitro against five human cancer cell lines: human non-small cell lung cancer cell line (A549), human melanoma cell line (A375), human colon cancer cell line (HCT116), human liver carcinoma cell line (HepG2), human breast adenocarcinoma cell line (MCF-7) by the Cell Counting Kit-8 (CCK-8) assay. Most of C-3 and C-28 MeON-neoglycosides of oleanolic acid exhibited notably inhibitory effects against the tested cancer cells and more sensitive to HepG2 cells than 5-Fluorouracil (5-FU). Structure-activities relationship (SAR) analysis revealed that sugar types and the d/l configuration of sugars would significantly affect their antiproliferative activities of neoglycosides. Among them, compound **8a** (28-*N*-methoxyaminooleanane-*β*-d-glucoside) exhibited the most potent antiproliferative activities against HepG2 cells with IC_50_ values of 2.1 µM. Further pharmacological experiments revealed that compound **8a** could cause morphological changes and cell cycle arrest at G0/G1 phase and induce apoptosis in HepG2 cells. These results suggested that neoglycosylation could provide a rapid strategy for the discovery of potential antiproliferative agents and their possible pharmacological mechanisms need more further research.

## 1. Introduction

Glycosylation is a naturally occurring process and many natural products derive their pharmacological properties such as target recognition, toxicity and even mechanism of action through this process [1,2]. Some anticancer drugs including azomycin, adriamycin and paclitaxel, can be specifically delivered to cancer cells by covalently conjugating them with carbohydrates [3]. Furthermore, the “Warburg effect” as a cellular phenomenon in cancer cells of displaying high rates of aerobic glycolysis with overexpression of glucose transporters such as GLUT1, provides clinically validated targets for cancer treatment [4,5]. Based on these fundamental insights, the design of glycoconjugates as various anticancer drugs becomes one of the important strategies in oncology research [5].

Oleanolic acid (3*β*-hydroxyolean-12-en-28-oic acid, OA), a natural pentacyclic triterpenoid, possesses many biological activities such as anti-inflammatory, anticancer and hepatoprotective effects and its natural derivates named saponins are bearing a glycan at either C-3 or C-28 via an ether or ester linkage respectively. Quite a number of these saponins have attracted much attention due to their remarkable broad spectrum of pharmacological activities [6], especially their antiproliferative activities [7,8,9]. For instance, camelliasaponin B1, which was isolated from the seed cake of *Camellia oleifera* Abel., exhibited broad potent antiproliferative activities against several human cancer cell lines (BEL-7402, BGC-823, MCF-7, HL-60) [10], proceraosides E-G, three new oleanane-type saponins, showed inhibitory activities against four human cancer cell lines with the IC_50_ values of 0.28–1.8 µM [11]. Therefore, glycosylation could be a practical strategy to expand and improve the biological activity of oleanolic acid.

Neoglycosylation is a mild chemoselective reaction between free reducing sugars and *N*-methoxyamino-substituted acceptors, which can produce the desired glycoconjugates of a selected target scaffold and thereby avoid the need for subsequent post-glycosylation modification/deprotection [12]. To date, a number of neoglycosides of natural products with potential anticancer activity against selective cancer lines were synthesized by neoglycosylation, such as perillyl alcohol [13], cyclopamine [14], and cardenolide [15,16,17]. In our previous work, we designed and synthesized four series of steroidal neoglycosides in D-ring and found that conjugation with the 2-deoxy-d-glucose could significantly enhance its anticancer activity, and compound **5k** ((25*R*)-3*β*-hydroxy-26-*N*-methoxyaminofurost-5-en-*β*-2-deoxy-d-glucoside) exhibited IC_50_ values even reaching 1.5 µM against HepG2 cells [18]. As a continuation of our research on the glycosylation of steroids and pentacyclic triterpenes, we conducted the neoglycosylaton of oleanolic acid on C-3 and C-28 and evaluated the cytotoxicity and selectivity on different cancer cell lines. However, it was difficult to direct neoglycosylation of oleanolic acid at C-3 due to the hindered adjacent C-4 dimethyl substitution. Therefore, a methoxyglycine handle was applied to distance the hindered dimethyl from the requisite neoglycosylation alkoxyamine. A series of C-3 and C-28 MeON-neoglycosides of oleanolic acid were designed and synthesized by neoglycosylation and their cytotoxic activity was evaluated against five human cancer cell lines by CCK-8 assay. Reducing sugars selected for this study included representative pentoses (d/l-ribose; d/l-arabinose; d/l-xylose; d/l-fucose; l-lyxose; l-rhamnose), hexoses (d/l-glucose; d-galactose; 3-O-methyl-d-glucose; d-mannose), 2-deoxy sugars (2-deoxy-d-glucose; 2-deoxy-d-galactose; 2-deoxy-d-ribose). Along with the neoglycosylation C-28-O-glucoside of oleanolic acid and erythrodiol were synthesized by biotransformation. After the cytotoxicity evaluation and structure-activity relationship (SAR) analsysis we selected the most potent compound for further pharmacological experiments including cell morphological changes, cell cycle arrest and apoptosis which could provide a better understand of the possible anti-proliferation mechanisms of neoglycosides.

## 2. Results and Discussion

### 2.1. Chemistry

#### 2.1.1. Synthesis of C-3 and C-28 MeON-neoglycosides of Oleanolic Acid by Neoglycosylation

**Scheme 1:**
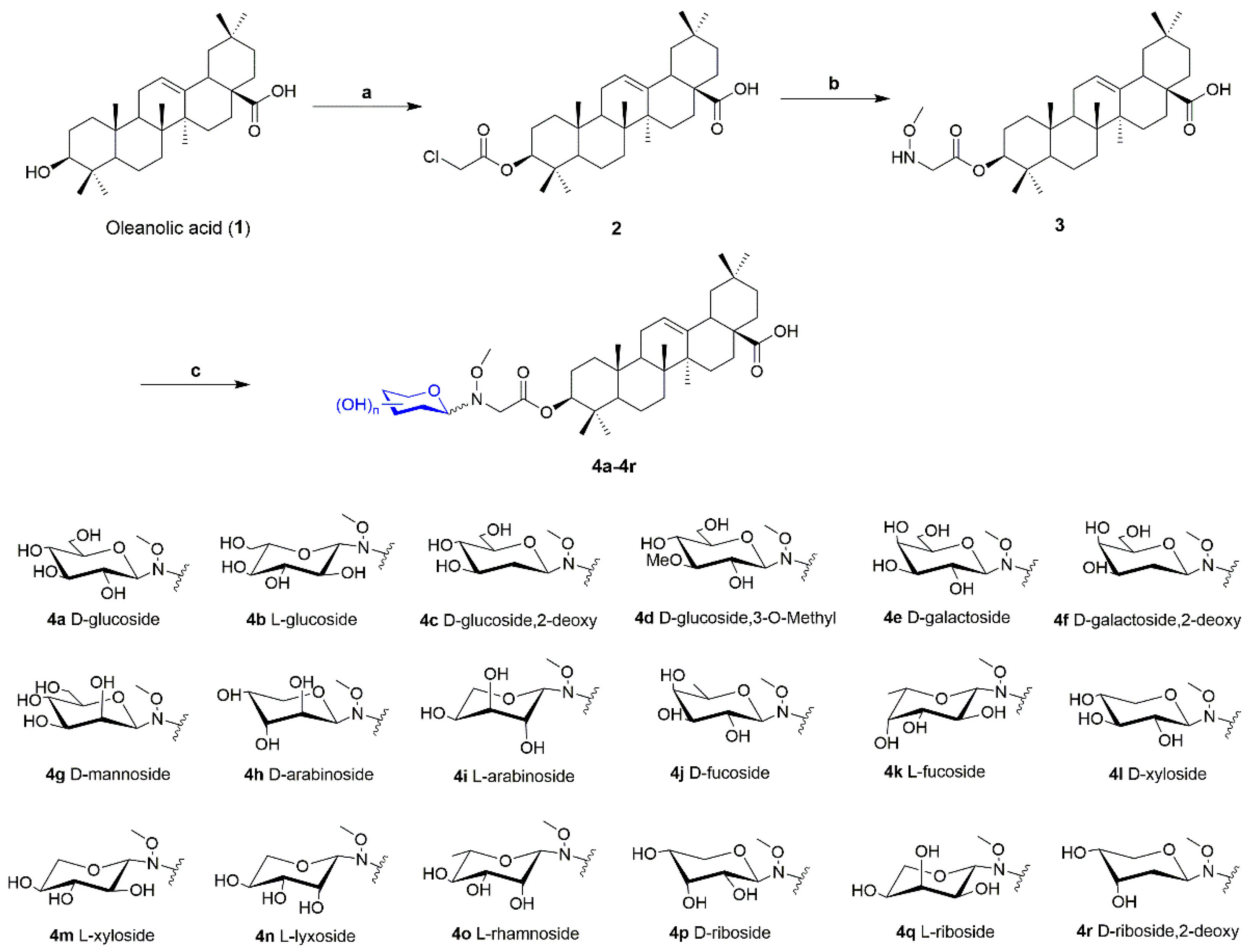
Synthesis of oleanolic acid C-3-neoaglycone **3** and neoglycosides **4a**–**4r**. *Reagents and conditions*: (**a**) ClCH_2_COCl, DIPEA, DMAP, THF, rt, 2 h; (**b**) NaI, EtOH, 60 °C, MeONH_2_ in THF, every 2 h; (**c**) reducing sugars, MeOH: CHCl_3_ (6:1), 40 °C, 48 h.

As depicted in Scheme 1, the preparation of oleanolic acid C-3-neoaglycone as the glycosyl acceptor was carried out according the reported literature method [19]. Oleanolic acid was esterified at the C-3 hydroxyl group using chloroacetyl chloride in the presence of DMAP. The chloride **2** was exchanged with iodide to facilitate the S_N_2 displacement by methoxyamine in the same reaction vessel. This two-step procedure provided neoaglycon **3** in 45% yield. It is unnecessary to add an external proton source because of the intrinsic carboxylic acid of **3**. Therefore, the glycosyl acceptor **3** was reacted with reducing sugars in MeOH/CHCl_3_ (6:1) at 40 °C for 48 h to obtain C-3 MeON-neoglycosides **4a–4r** [19].

**Scheme 2:**
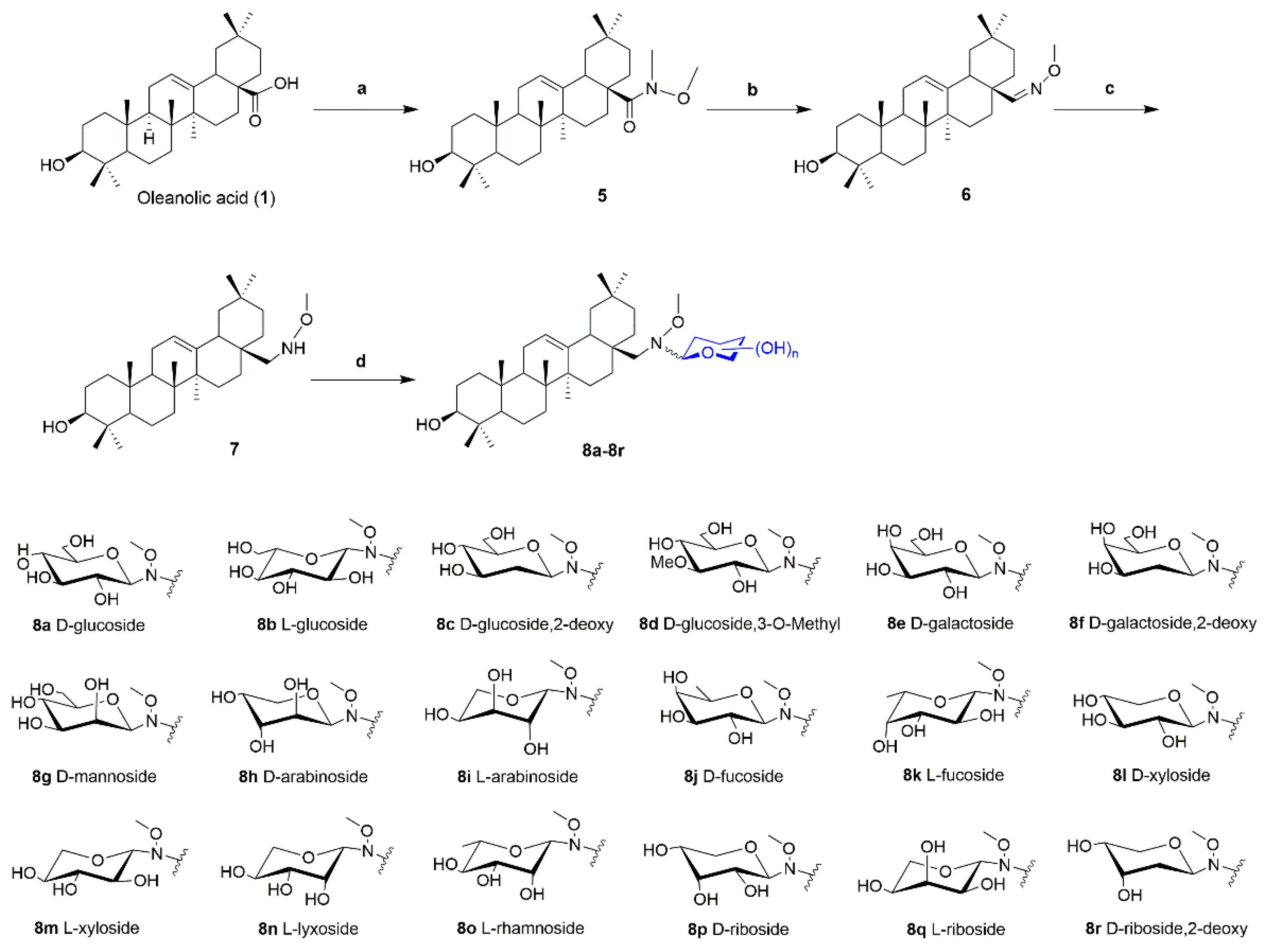
Synthesis of oleanolic acid C-28-neoaglycone **7** and neoglycosides **8a**–**8r**. *Reagents and conditions*: (**a**) MeON(H)Me, NMM, EDAC, 0 °C, 2 h; (**b**) LAH, THF; CH_3_ONH_2_·HCl, Pyridine, MeOH: CH_2_Cl_2_ (4:1), reflux, 55 °C, 8 h; (**c**) NaCNBH_3_, AcOH, rt, 10 h; (**d**) reducing sugars, MeOH: CHCl_3_ (4:1), AcOH, 40 °C, 48 h.

The aldehyde or ketone group is an adaptive condition for neoglycosylation reactions, herein the synthesis of oleanolic acid C-28-neoaglycone **7** was initiated by preparation of C-28-oleanolic acid aldehyde (Scheme 2). Briefly, oleanolic acid was converted into the Weinreb amide **5** using the coupling agent EDAC, and then selectively reduced to the corresponding aldehyde with LAH. The resulting compound was subsequently condensed with methoxyamine hydrochloride in the presence of organic base to obtain compound **6**. Then, reduction of the carbon-nitrogen double bond was accomplished withNaCNBH_3_ in the presence of acetic acid to afford the requisite oleanolic acid C-28-neoaglycone **7**. The glycosyl acceptor **7** was reacted with reducing sugars in solvent system (MeOH/CHCl_3_, 4:1) and external proton source (acetic acid, 10 eq.) at 40 °C for 48 h to obtain C-28 MeON-neoglycosides **8a**–**8r** based on our previous study [20]. The glycosidic bond configuration of all the C-3 and C-28 MeON-neoglycosides was characterized according to the value of coupling constant of anomeric positions. However, it is worth mentioning that oleanolic acid neoglycosides presented highly selective for d/l-glucose, d/l-xylose to form *β*-anomer of neoglycosides (Supporting Information Table S1 and Figure S1). This anomeric stereoselectivity in the glycosidation process may be attributed to the thermodynamics and the stereochemistry of C-2. Peri observed C-2-equatorial glycosides (e.g., glucose, galactose) preferring the *b*-anomer and C-2-axial glycosides (e.g., mannose) with the a-configuration due to a thermodynamic equilibrium between the open iminium intermediate and closed ring form [21]. It was significantly inclined to the *b*-anomer for C-2-equatorial glycosides in neoglycoside libraries [22]. In the neoglycoside synthesis, the solvent system may all greatly affect the yields, solubility equilibrium between the reducing sugars and the aglycones is very essential for the whole process. Polar aprotic solvents (DMSO, DMF) are frequently used [23], but evaporating DMF from crude reaction mixtures would significantly diminish the efficiency of the reaction and the MeOH/CHCl_3_ system in this experiment also resulted in variable and considerable yields.

#### 2.1.2. Synthesis of C28-O-Glycosides of Oleanolic Acid, Erythrodiol by Biotransformation

**Scheme 3:**
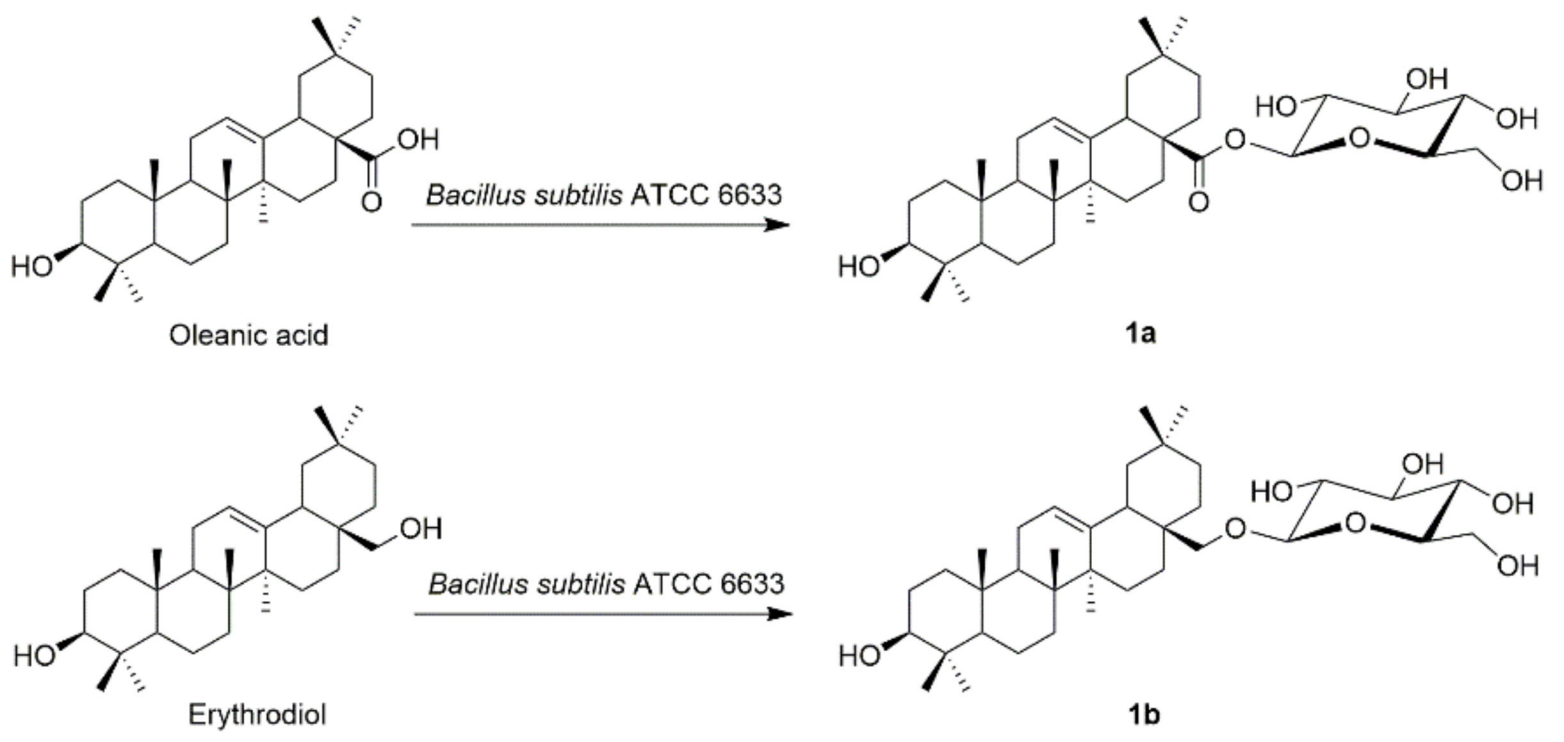
Biotransformation of oleanolic acid, erythrodiol by *Bacillus subtilis* ATCC 6633.

Biotransformation is a method of structural modification by a microbial enzyme system which facile and green in one step. Our research group has been committed to the biotransformation of pentacyclic triterpenes and found that the *Bacillus subtilis* ATCC 6633 possessed a high glycosylation capability. Herein we tested the glycosylation capability of *Bacillus subtilis* ATCC 6633 to oleanolic acid and erythrodiol, one more polar metabolite is detected and then isolated and identified as **1a** (oleanolic acid-28-O-*β*-D-glucopyranoside) [24], **1b** (erythrodiol-28-O-*β*-D-glucopyranoside) [25], respectively (Scheme 3).

### 2.2. Antiproliferative Activity Evaluation

The cytotoxic activities of C-3 and C-28 MeON-neoglycosides of oleanolic acid were evaluated against the tested cell lines (A549, HepG2, MCF-7, A375, HCT116) using Cell Counting Kit-8 (CCK-8) assay, with 5-Fluorouracil (5-FU) as a reference.

As shown in Table 1 and Table 2, compared with the positive control 5-FU, most of neoglycosides, either neoglycosylation on C-3 or C-28, exhibited considerable inhibitory effects against the tested cancer cells. Among them, the sugar types had notably influences on their antiproliferative activity, compounds with the d-glucose (**4a**, **8a**) and d-galactose (**4e**, **8e**) displayed notably antiproliferative activities and more sensitive to HepG2 cells. Especially compound **8a** with the d-glucose exhibited the most potent and selective growth inhibition against HepG2 cells with IC_50_ value of 2.1 µM which was consistent with the hepatoprotective activity of oleanolic acid on liver cells. In our previous steroid neoglycosylation research, the streroidal neoglycosides with 2-deoxy sugars showed significantly enhanced antiproliferative activities against the tested cancer cell lines [20] which indicated that in the neoglycosylation different sugars conjugated on different aglycons may vary their contribution of antiproliferative effects. Therefore, these findings would provide us some useful information to identify more potent anticancer agents for the subsequent research on pentacyclic triterpenes neoglycosylation. 

The sugar configuration also had significantly influences on their antiproliferative activity. When comparing the antiproliferative activities of compounds **4a**/**4b**, **4h**/**4i**, **4j**/**4k**, **4l**/**4m** and **4p**/**4q**, we found that compounds with d-sugars (**4a**, **4h**, **4j**, **4p**) showed more potent antiproliferative activities than correspond compounds with l-sugars (**4b**, **4i**, **4k**, **4q**) except the xylose C-3 MeON-neoglycosides (Table 1). Similarly, in C-28 MeON-neoglycosides group compounds with the d-glucose (**8a**), d-arabinose (**8h**) displayed also stronger antiproliferative activities against the tested cells than with the l-glucose (**8b**), l-arabinose (**8i**) (Table 2) while compounds with l-fucose (**8k**) and l-ribose (**8q**) displayed stronger antiproliferative activities against the tested cells than the compounds with the d-fucose (**8j**) and d-ribose (**8p**). And compound **8m** with the l-xylose presented significant antiproliferative activity than compound **8l** with the d-xylose (Table 2). 

It was noteworthy that for the same sugar, different linked sites could also affect their antiproliferative activities. In C-28 MeON-neoglycosides group compounds with l-arabinose (**8i**), d-fucose (**8j**), d-xylose (**8l**), l-lyxose (**8n**) showed no effects against the tested cells at the concentration of 30 µM, while compounds with l-arabinose (**4i**), d-fucose (**4j**), d-xylose (**4l**), l-lyxose (**4n**) in C-3 MeON-neoglycosides group exhibited distinctly antiproliferative activities. In addition, the antiproliferative activity of the potent compound **8a** (28-*N*-Methoxyaminooleanane-β-d-glucoside) also exhibited stronger anticancer effects than natural C-28-O-glucoside of oleanolic acid (**1a**) and erythrodiol (**1b**) (Table 2). 

From above results, we can find that most of these MeON-neoglycosides exhibited significantly higher antiproliferative activities against the tested cell lines than 5-FU (Table 1). It was also intriguing that HepG2 cells was more sensitive to these compounds than other originated cancer lines. Among them, compound **8a** was the most potent one against HepG2 cells with the IC_50_ value of 2.1 µM. It proved once again that sugar type, sugar configuration and sugar conjugate site were the three key factors of neoglycosylation which would significantly affect their antiproliferative activities.

### 2.3. Morphological Changes of HepG2 Cells Induced by Compound **8a**

**Figure 1:**
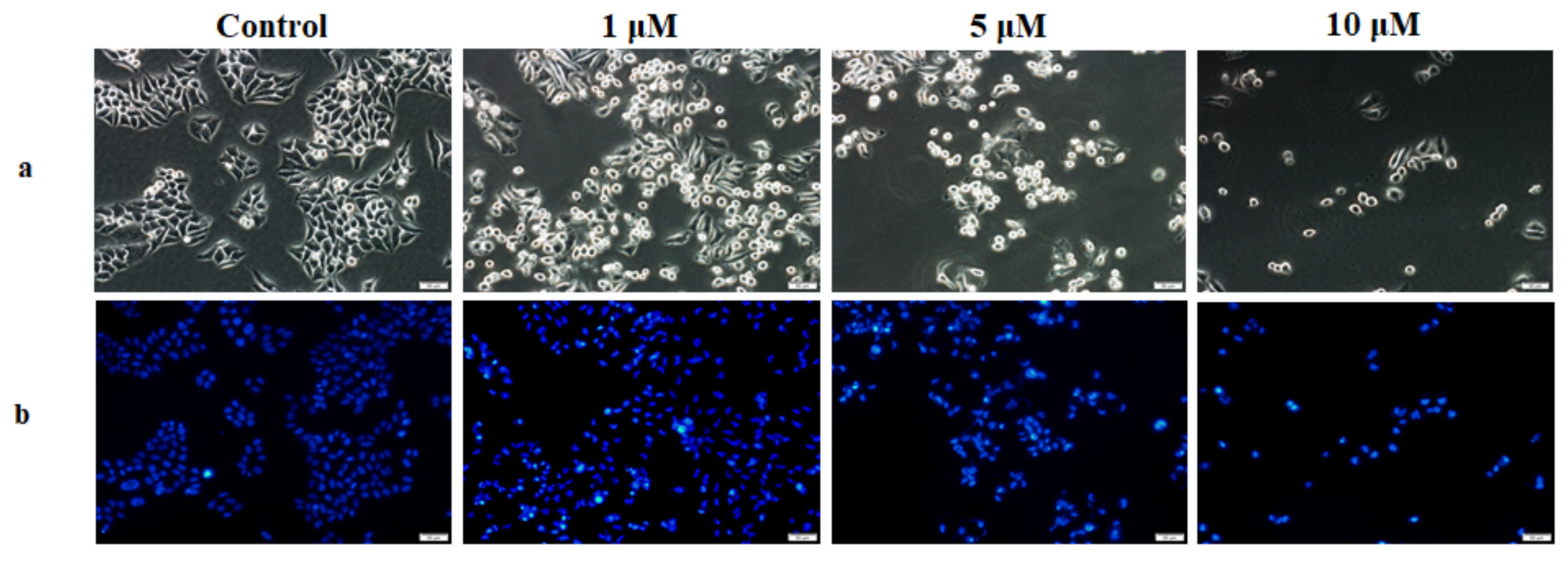
HepG2 cells morphological changes (**a**) and Hoechst 33342 staining (**b**) after treated with compound **8a** (1, 5, 10 µM) for 24 h. Scale bar, 50 μm.

Morphological changes of cancer cells are always associated with the growth inhibition induced by cytotoxic agents [26]. After being incubated with **8a** for 24 h at different concentrations (1, 5, 10 µM), the morphological changes of HepG2 cells were recorded using an inverted microscope. Compared with the control group, some of the **8a**-treated cells exhibited rounding, shrinkage, membrane blebbing, especially at high concentrations (Figure 1a). Hoechst 33342 staining was used to assess nuclear changes in HepG2 cells. We found that the chromatin is markedly shrunk after incubation with compound **8a** for 24 h (Figure 1b).

### 2.4. Compound **8a** Caused Cell Cycle Arrest at the G0/G1 Phase in HepG2 Cells

**Figure 2:**
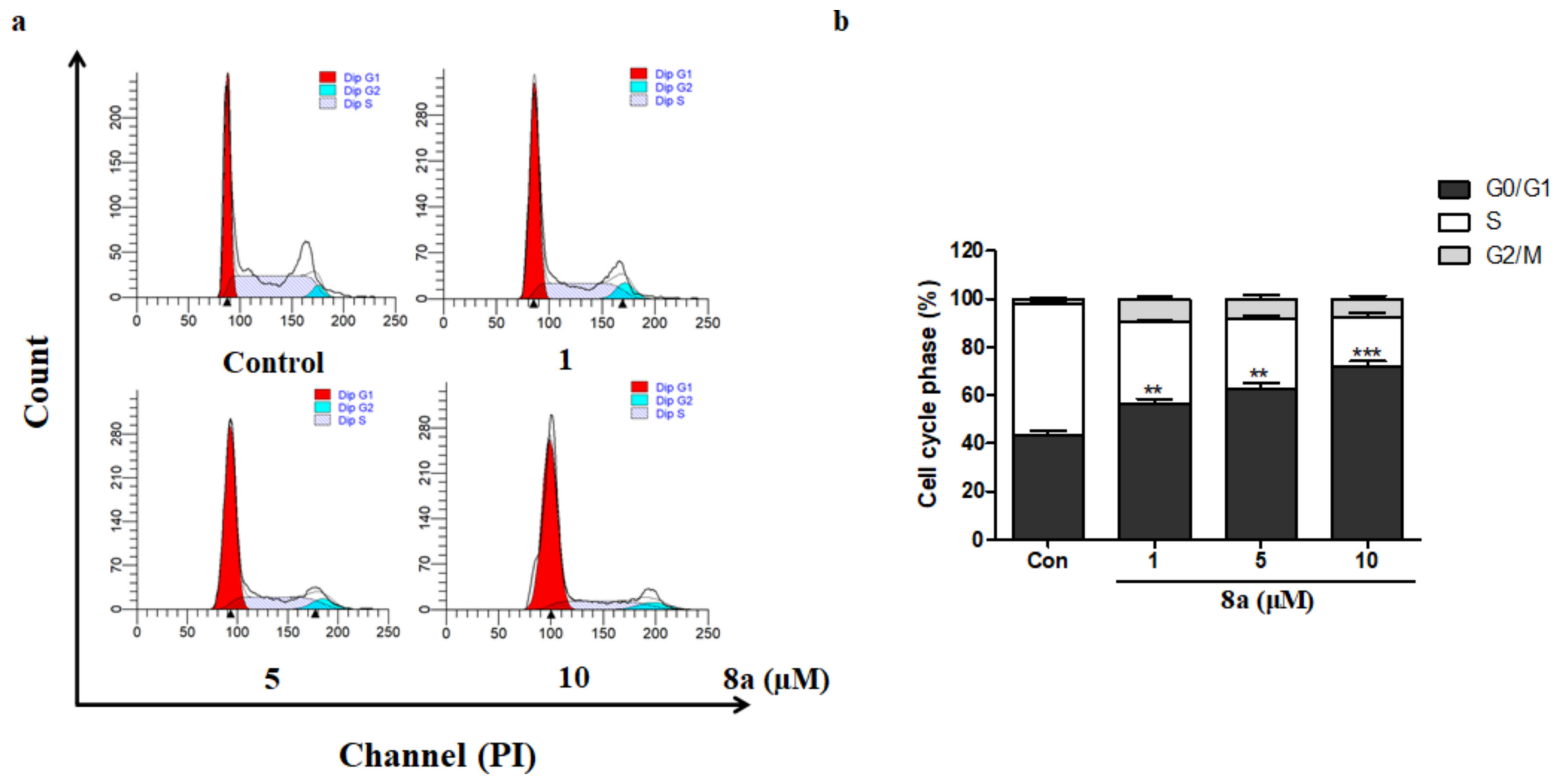
Compound **8a** induces G0/G1 arrest in HepG2 cells. (**a**). Flow cytometric plots show the cell cycle distribution when the cells were treated with **8a** (1, 5, 10 µM) for 24 h; (**b**). Bar graphs show the cell cycle distribution when the cells were treated with compound **8a**; ** *p* < 0.01, *** *p* < 0.001 vs. the control group. All data are expressed as mean ± SD of the three independent experiments.

Inducing cells cycle arrest constitutes one of the most prevalent strategies used to prevent cancer development [27]. To establish whether compound **8a** could inhibit the cell growth by interrupting the cell cycle progression, cellular DNA was analyzed by flow cytometry using propidium iodide (PI) staining. The profiles were shown in Figure 2a. Obviously, compared with the control group, the G0/G1 population of HepG2 cells was increased after treatment with **8a** from 43.53% (0 µM) to 56.12% (1 µM), 62.75% (5 µM) and 71.69% (10 µM). These results indicated that compound **8a** could induce cell cycle arrest of HepG2 cells at G0/G1 phase.

### 2.5. Compound **8a** Induced Apoptosis in HepG2 Cells

**Figure 3:**
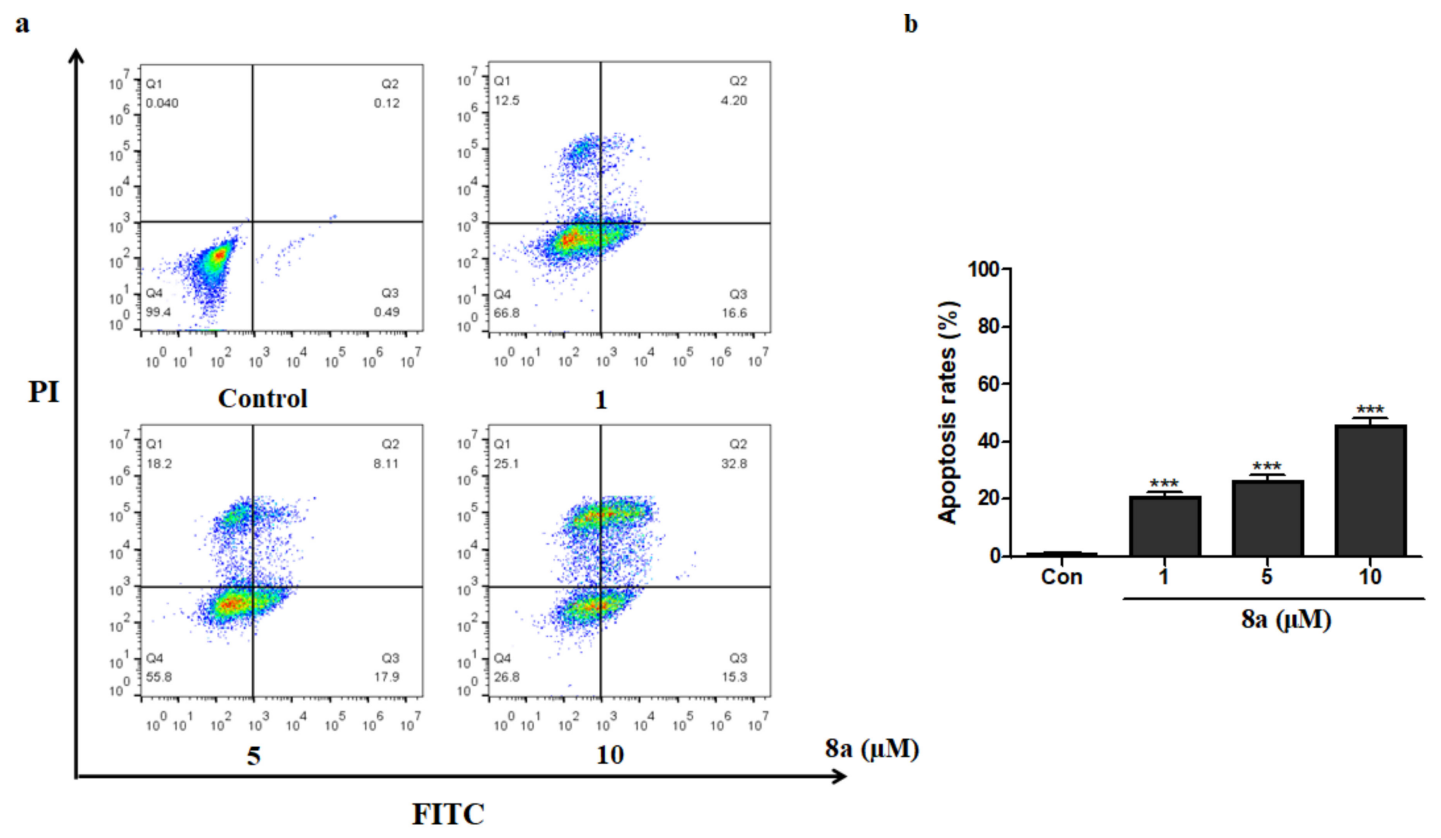
Compound **8a** induces apoptosis in HepG2 cells. (**a**). Flow cytometric plots show cells in the different stages when the cells were treated with compound **8a** (1, 5, 10 µM) for 24 h; (**b**). Bar graphs show the percentage of apoptosis cell populations when the cells were treated with **8a**; *** *p* < 0.001 vs. the control group. All data are expressed as mean ± SD of the three independent experiments.

Apoptosis was generally considered as the predominant form of regulated cell death responsible for cancer therapies [28]. In order to test whether the compound **8a** could induce apoptosis, the percentage of apoptotic cells was determined by flow cytometry following Annexin V-FITC and propidium iodide (PI) double staining. A dose-dependent increase in the percentage of apoptotic cells was noted after the cells were treated for 24 h with compound **8a** at 1 µM, 5 µM, 10 µM. As shown in Figure 3a, very few (0.61%) apoptotic cells were present in the control panel, whereas the percentage of apoptotic cells significantly increased to 48.1% in neoglycoside **8a**-treated group. These results indicate that compound **8a** was a potential cancer cells apoptosis inducer.

At present, there are few studies on the anticancer mechanisms of neoglycosides. New inverse molecular docking protocol was recently proposed to identify potential human protein targets of natural products, its predictions were in agreement with the scientific literature and confirmed that curcumin binds to folate receptor *β*, DNA (cytosine-5)-methyltransferase 3A, metalloproteinase-2, mitogen-activated protein kinase 9, epidermal growth factor receptor and apoptosis-inducing factor 1 [29]. While many studies clearly illustrated that the conjugation of sugars were critical for altering both the mechanism-of-action and potency of the parent drug, determination of the precise anticancer mode-of-action of neoglycosides remains to be challenging. Therefore, we will continue to employ the inverse molecular docking tool to explore the potential mechanisms for neoglycosides in the treatment of cancers. 

## 3. Materials and Methods

### 3.1. Materials and Instruments

All starting materials and reagents were obtained from commercial suppliers as follows: All reducing sugars were purchased from Shanghai Yuanye Bio-Technology Co., Ltd. (Shanghai, China). Oleanolic acid was purchased from Nanjing jingzhu Bio-Technology Co., Ltd. (Nanjing, China). Methoxylamine hydrochloride (CH_3_ONH_2_·HCl), sodium cyanoborohydride (NaCNBH_3_), 4-dimethylaminopyridine (DMAP), *N,N*-diisopropylethylamine (DIPEA) were purchased from Shanghai Saen Chemical Technology Co., Ltd. (Shanghai, China). Reaction progress was monitored by analytical TLC on 0.50 mm Silica Gel 60 F254 plates (Qingdao Ocean Chemical Factory, Qingdao, Shandong, China) and observed by spraying with 10% ethanol sulfate solution. ^1^H- and ^13^C-NMR spectra were obtained on an AV-400 or AV-500 spectrometer (Bruker, Germany). The chemical shifts of ^1^H and ^13^C were referenced to TMS (for CDCl_3_) or (for C_5_D_5_N). Multiplicities was represented by s (singlet), d (doublet), t (triplet), q (quartet) and m (multiplet). Chemical shift assignments for anomeric mixtures, where possible, are noted as *α* or *β* with the atom responsible for the shift. Mass spectrometric data were recorded on a 6530 QTOF spectrometer (Agilent, Santa Clara, CA, USA) using electrospray ionization. 

### 3.2. Chemistry

#### 3.2.1. Synthesis of (3S)-O-Chloroacetyloleanolic Acid (**2**)

Oleanolic acid (**1**, 2.1 g, 4.5 mmol) and DMAP (54 mg, 0.42 mmol) were dissolved in anhydrous THF (40 mL) under nitrogen. Diisopropylethylamine (DIPEA, 1.14 mL, 6.54 mmol) was added followed by dropwise addition of chloroacetyl chloride (0.96 mL, 12.1 mmol). After stirring for 2 h, absolute ethanol (1.5 mL) was used to quench the reaction. The solvent was removed in vacuo and the resulting crude product was adsorbed onto silica gel, after dissolving in CH_2_Cl_2_ (20 mL), then purified by column chromatography to obtain the desired chloroacetate as a white solid **2** (2.13 g, 87%). ^1^H-NMR (400 MHz, CDCl_3_) δ 5.24 (t, *J* = 3.4 Hz, 1H), 4.69–4.50 (m, 1H), 4.12 (s, 1H), 4.05 (d, *J* = 2.4 Hz, 2H), 2.24–1.18 (m, 24H), 1.08 (s, 3H), 1.00–0.93 (m, 7H), 0.89 (s, 3H), 0.86 (d, *J* = 7.9 Hz, 6H), 0.77 (s, 3H). ^13^C-NMR (100 MHz, CDCl_3_) δ 183.7, 167.3, 138.1, 125.9, 83.5, 55.4, 52.7, 48.1, 47.6, 42.1, 41.4, 39.6, 39.2, 39.0, 38.3, 38.1, 37.0, 36.8, 33.0, 33.0, 30.7, 28.2, 28.1, 24.2, 23.7, 23.6, 23.4, 21.3, 18.3, 17.1, 16.8, 15.7. HRMS (ESI) *m*/*z* for C_32_H_48_ClO_4_ ([M-H]^−^) 531.3251, calc. 531.3247.

#### 3.2.2. Synthesis of (3S)-O-(N-Methoxyglycyl) Oleanolic Acid (**3**)

Chloroacetate **2** (2 g, 3.75 mmol) was dissolved in absolute ethanol (160 mL) along with NaI (1.82 g, 12.15 mmol) under nitrogen. After stirring at room temperature for 2 h, a solution of MeONH_2_ in THF (2.4 M, 1.9 mL, 4.56 mmol; made by stirring CH_3_ONH_2_·HCl in a NaOH/THF slurry for 24 h) was added, the nitrogen line removed, the reaction mixture was heated to 60 °C and stirred for 2 h, then the reaction was cooled to room temperature and another aliquot of MeONH_2_ in THF (2 eq.) was introduced followed by reheating to 60 °C. This additive process was repeated roughly every 2 h until the reaction had progressed sufficiently based upon TLC which occurred after ~24 h of total reaction time. The solvent was removed and the product purified by column chromatography (0.92 g, 45%). ^1^H-NMR (500 MHz, C_5_D_5_N) δ 5.49 (s, 1H), 4.93–4.63 (m, 1H), 3.92 (s, 2H), 3.63 (s, 3H), 3.32 (d, *J* = 11.3 Hz, 1H), 1.30 (s, 3H), 1.03 (s, 3H), 1.01 (s, 3H), 0.98 (s, 3H), 0.96 (s, 3H), 0.91 (s, 3H), 0.84 (s, 3H). ^13^C-NMR (125 MHz, C_5_D_5_N) δ 180.5, 171.6, 145.2, 122.7, 81.7, 61.8, 55.9, 54.2, 48.3, 47.0, 46.8, 42.5, 42.4, 40.1, 38.5, 38.4, 37.5, 34.6, 33.7, 33.6, 33.4, 31.4, 28.7, 28.5, 26.6, 24.3, 24.2, 24.2, 24.1, 18.9, 17.7, 17.3, 15.7. HRMS (ESI) *m*/*z* for C_33_H_52_NO_5_ ([M-H]**^−^**) 542.3851, calc. 542.3851.

#### 3.2.3. Synthesis of (3β)-Hydroxy-*N*-methyl-*N*-methoxy-olean-12-en-28-amide (**5**)

Oleanolic acid (**1**, 2.75 g, 5 mmol) was dissolved in anhydrous CH_2_Cl_2_ (200 mL), stirred and ice-bathed for 30 min. Then *N,O*-dimethylhydroxylamine hydrochloride (0.6 g, 6 mmol) and *N*-methylmorpholine (0.8 mL, 6 mmol) were added in order. The 1-(3-dimethylaminopropyl)-3-ethylcarbodiimide hydrochloride (EDAC, 1.5 g, 6.25 mmol) was slowly added over 10 min. After reacting in an ice-bath for 2 h, 5% HCl aqueous solution (50 mL) was added to quench the reaction. The acidic aqueous layer was extracted with CH_2_Cl_2_ (3 × 100 mL). The organic layers were combined and washed with saturated NaHCO_3_ solution and saturated NaCl solution, respectively. The organic layer was dried over anhydrous Na_2_SO_4_, then purified by column chromatography to obtain the desired white solid **5** (79%, 2.4 g). ^1^H-NMR (500 MHz, C_5_D_5_N) δ 5.43 (t, *J* = 3.4 Hz, 1H), 3.62 (s, 3H), 3.44 (d, *J* = 6.1 Hz, 1H), 3.34 (dd, *J* = 13.7, 3.9 Hz, 1H), 3.20 (s, 3H), 1.26 (s, 3H), 1.25 (s, 3H), 1.04 (s, 3H), 0.96 (s, 3H), 0.93 (s, 9H). ^13^C-NMR (125 MHz, C_5_D_5_N) δ 178.4, 145.3, 122.4, 78.2, 60.4, 56.0, 48.3, 48.1, 46.8, 42.5, 42.3, 39.7, 39.5, 39.0, 37.5, 34.4, 34.2, 33.3, 33.3, 30.8, 29.6, 28.9, 28.6, 28.2, 26.4, 23.9, 23.9, 22.5,18.9, 17.4, 16.6, 15.7. HRMS (ESI) *m*/*z* for C_32_H_54_NO_3_ ([M+H]^+^) 500.4090, calc. 500.4098.

#### 3.2.4. Synthesis of (3β)-Hydroxy-28-*N*-methyloxime-olean-12-en (**6**)

Lithium aluminium hydride (LAH, 0.6 g, 5 eq) was dissolved in anhydrous THF (100 mL) under nitrogen. After in an ice-bath under nitrogen for 30 min, a solution of compound **5** in THF (1.5 g, dissolved in 30 mL anhydrous THF) was added dropwise. After another 30 min of ice-bath reaction, the reaction continued at room temperature for 4 h. The reaction was quenched with saturated NH_4_Cl solution, and the aqueous layer extracted with EtOAc (3 × 100 mL). The combined organic layer was washed with saturated NaCl solution, and then was dried over anhydrous Na_2_SO_4_. The crude product was redissolved with MeOH/CH_2_Cl_2_ (4:1, 150 mL), followed by addition of methoxyamino hydrochloride (CH_3_ONH_2_·HCl, 5 eq.) and pyridine (5 eq.), respectively. The reaction was heated to 55 °C under reflux for 8 h. Then purified by column chromatography to obtain the desired compound as a white solid **6** (81%, 1.1 g). ^1^H-NMR (500 MHz, C_5_D_5_N) δ 5.30 (t, *J* = 3.5 Hz, 1H), 3.92 (s, 3H), 3.48–3.40 (m, 1H), 2.52 (dd, *J* = 13.6, 4.0 Hz, 1H), 1.25 (s, 3H), 1.21 (s, 3H), 1.05 (s, 3H), 0.97 (s, 3H), 0.96 (s, 3H), 0.90 (s, 3H), 0.89 (s, 3H). ^13^C-NMR (125 MHz, C_5_D_5_N) δ 158.7, 144.4, 123.4, 78.3, 61.4, 56.0, 48.3, 46.3, 44.4, 42.1, 40.4, 39.9, 39.6, 39.2, 37.5, 34.2, 33.4, 33.3, 33.3, 31.0, 29.0, 28.3, 27.2, 26.5, 24.9, 24.0, 23.8, 19.0, 17.7, 16.8, 15.8. HRMS (ESI) *m*/*z* for C_31_H_52_NO_2_ ([M+H]^+^) 470.3991, calc. 470.3993.

#### 3.2.5. Synthesis of (3β) 3-Hydroxy-28-*N*-methoxy-olean-12-en (**7**)

Compound **6** (1 g, 2.1 mmol) was dissolved in acetic acid (100 mL), NaCNBH_3_ (10 eq.) was added. The reaction was stirred at room temperature for 10 h. Quench the reaction by adding saturated NaHCO_3_ solution (100 mL), and then was extracted with CH_2_Cl_2_ (3 × 100 mL). The organic layer was washed with saturated NaHCO_3_ solution, saturated NaCl solution, and was dried over anhydrous Na_2_SO_4_. Filtered, and the solvent was condensed under reduced pressure. The crude product was purified by column chromatography with PE/EtOAc (10: 1) to obtain desired product as a white solid **7** (83%, 0.83 g). ^1^H-NMR (500 MHz, C_5_D_5_N) δ 5.25 (t, *J* = 3.4 Hz, 1H), 3.63 (s, 3H), 3.43 (d, *J* = 9.0 Hz, 1H), 3.25 (dd, *J* = 11.7, 8.5 Hz, 1H), 2.77 (dd, *J* = 11.8, 6.8 Hz, 1H), 2.17 (dd, J = 13.5, 3.8 Hz, 1H), 1.24 (s, 3H), 1.23 (s, 3H), 1.04 (s, 3H), 1.01 (s, 3H), 0.94 (s, 3H), 0.91 (s, 3H), 0.90 (s, 3H). ^13^C-NMR (125 MHz, C_5_D_5_N) δ 145.0, 122.9, 78.2, 61.3, 59.9, 55.9, 48.2, 46.8, 45.2, 42.0, 40.4, 39.6, 39.3, 37.4, 36.1, 34.7, 33.6, 33.2, 33.1, 31.3, 28.9, 28.3, 26.4, 26.3, 24.1, 23.9, 23.5, 19.0, 17.2, 16.8, 16.0. HRMS (ESI) *m*/*z* for C_31_H_54_NO_2_ ([M+H]^+^) 472.4142, calc. 472.4149.

#### 3.2.6. General Procedure for the Synthesis of Oleanolic Acid Neoglycosides **4a**–**4r** and **8a**–**8r**

To a solution of neoaglycone **3** (typically 0.1 mmol, 54.2 mg) and reducing sugar (2 eq.) were dissolved in MeOH*/*CHCl_3_ (6:1, 5 mL). Neoaglycon **7** (0.1 mmol, 47.2 mg) and reducing sugar (2 eq.) was dissolved in MeOH*/*CHCl_3_ (4:1, 5 mL), external proton source AcOH (10 eq.) were added, and then reaction at 40 °C for 48 h on a rotary shaker at 250 rpm. The target neoglycosides was purified with MeOH/CH_2_Cl_2_ by silica gel column chromatography. The configuration of the glycosidic bond of all the neoglycosides was identified by the *J* value of *J*_H1′_−_H2′_.

*(3S)-O-(N-Methoxy-N-d-glucosylglycyl) oleanolic acid* (**4a**) 

White solid (15.2 mg, 21%). ^1^H-NMR (500 MHz, C_5_D_5_N) δ 5.50 (t, *J* = 3.3 Hz, 1H), 4.86–4.77 (m, 2H), 4.58 (dd, *J* = 11.9, 2.2 Hz, 1H), 4.43–4.33 (m, 2H), 4.32–4.19 (m, 3H), 4.15 (d, *J* = 16.9 Hz, 1H), 4.05 (s, 3H), 4.00–3.94 (m, 1H), 3.33 (dd, *J* = 13.8, 4.0 Hz, 1H), 1.31 (s, 3H), 1.04 (s, 3H), 1.01 (s, 3H), 0.98 (s, 3H), 0.89 (s, 3H), 0.84 (s, 3H), 0.82 (s, 3H). ^13^C-NMR (125 MHz, C_5_D_5_N) δ 180.5, 170.7, 145.2, 122.8, 95.6, 81.4, 80.8, 80.2, 72.1, 71.9, 63.3, 62.8, 55.9, 55.7, 48.3, 47.0, 46.8, 42.5, 42.4, 40.1, 38.6, 38.3, 37.5, 34.6, 33.6, 33.6, 33.4, 31.3, 28.7, 28.5, 26.6, 24.3, 24.1, 24.1, 24.1, 18.8, 17.7, 17.3, 15.8. HRMS (ESI) *m*/*z* for C_39_H_64_NO_10_ ([M+H]^+^) 706.4525, calc. 706.4525.

*(3S)-O-(N-Methoxy-N-β-l-glucosylglycyl) oleanolic acid* (**4b**) 

White solid (11.0 mg, 16%). ^1^H-NMR (500 MHz, C_5_D_5_N) δ 5.50 (t, *J* = 3.1 Hz, 1H), 4.84 (d, *J* = 8.7 Hz, 1H), 4.81 (dd, *J* = 11.2, 5.4 Hz, 1H), 4.57 (dd, *J* = 11.8, 2.1 Hz, 1H), 4.40 (dd, *J* = 11.9, 5.4 Hz, 1H), 4.35–4.12 (m, 5H), 4.05 (s, 3H), 4.00–3.93 (m, 1H), 3.33 (dd, *J* = 13.7, 3.9 Hz, 1H), 1.30 (s, 3H), 1.04 (s, 3H), 1.01 (s, 3H), 0.98 (s, 3H), 0.92 (s, 3H), 0.84 (s, 3H), 0.83 (s, 3H). ^13^C-NMR (125 MHz, C_5_D_5_N) δ 180.0, 170.2, 144.7, 122.2, 94.9, 80.8, 80.2, 79.6, 71.6, 71.3, 62.7, 62.1, 55.3, 54.9, 47.7, 46.5, 46.3, 42.0, 41.8, 39.5, 38.0, 37.8, 37.0, 33.8, 33.1, 33.0, 32.8, 31.3, 28.1, 27.9, 26.0, 23.7, 23.6, 23.5, 22.7, 18.3, 17.2, 16.6, 15.2. HRMS (ESI) *m*/*z* for C_39_H_64_NO_10_ ([M+H]^+^) 706.4533, calc. 706.4525.

*(3S)-O-(N-Methoxy-N-(2-deoxy-d-glucosyl)glycyl) oleanolic acid* (**4c**) 

White solid (38.5 mg, 56%). ^1^H-NMR (500 MHz, C_5_D_5_N) δ 5.49 (s, 1H), 4.83–4.77 (m, 2H), 4.55 (dd, *J* = 11.8, 2.3 Hz, 1H), 4.45–3.99 (m, 5H), 3.88 (s, 3H), 3.86–3.82 (m, 1H), 3.32 (dd, *J* = 13.7, 3.8 Hz, 1H), 2.71–2.54 (m, 1H), 1.30 (s, 3H), 1.03 (s, 3H), 1.01 (s, 3H), 0.98 (s, 3H), 0.92 (s, 3H), 0.85 (d, *J* = 4.0 Hz, 6H). ^13^C-NMR (125 MHz, C_5_D_5_N) δ 179.9, 170.0, 144.7, 122.2, 91.0, 80.9, 80.3, 73.2, 73.0, 62.8, 62.4, 55.5, 55.3, 47.7, 46.5, 46.3, 42.0, 41.8, 39.5, 38.0, 37.8, 37.5, 37.0, 34.0, 33.1, 33.0, 32.8, 30.8, 28.1, 27.9, 26.0, 23.7, 23.6, 23.6, 23.5, 18.3, 17.2, 16.8, 15.2. HRMS (ESI) *m*/*z* for C_39_H_64_NO_9_ ([M+H]^+^) 690.4575, calc. 690.4576.

*(3S)-O-(N-Methoxy-N-(3-O-methyl-d-glucosyl)glycyl) oleanolic acid* (**4d**) 

White solid (10.7 mg, 15%). ^1^H-NMR (500 MHz, C_5_D_5_N) δ 5.48 (s, 1H), 4.89–4.70 (m, 2H), 4.56–4.29 (m, 5H), 4.26–4.05 (m, 2H), 4.02 (s, 3H), 3.96 (s, 3H), 3.93–3.84 (m, 1H), 3.32 (d, *J* = 13.2 Hz, 1H), 1.30 (s, 3H), 1.02 (d, *J* = 9.6 Hz, 3H), 1.00 (d, *J* = 4.8 Hz, 3H), 0.98 (d, *J* = 2.4 Hz, 3H), 0.88 (s, 3H), 0.84 (s, 3H), 0.82 (d, *J* = 3.8 Hz, 3H). ^13^C-NMR (125 MHz, C_5_D_5_N) δ 180.5, 168.2, 145.2, 122.7, 95.5, 82.0, 81.5, 80.6, 71.7, 71.1, 66.0, 63.0, 62.7, 55.9, 55.8, 48.3, 47.0, 46.8, 42.5, 42.3, 40.1, 38.5, 38.3, 37.5, 34.6, 33.6, 33.6, 33.4, 31.2, 28.7, 28.5, 26.5, 24.3, 24.1, 24.0, 23.3, 19.8, 17.7, 17.3, 14.1. HRMS (ESI) *m*/*z* for C_40_H_66_NO_10_ ([M+H]^+^) 720.4678, calc. 720.4681.

*(3S)-O-(N-Methoxy-N-d-galactosylglycyl) oleanolic acid* (**4e**) 

White solid (16.1 mg, 23%). ^1^H-NMR (500 MHz, C_5_D_5_N) δ 5.50 (d, *J* = 11.1 Hz, 1H), 5.34 (d, *J* = 5.7 Hz, 0.67H, αH1′), 4.87–4.81 (m, 0.67H), 4.78 (dd, *J* = 10.4, 5.7 Hz, 1H), 4.63–4.43 (m, 2H), 4.42–4.13 (m, 4H), 3.99 (s, 3H), 3.95–3.89 (m, 1H), 3.44–3.25 (m, 1H), 1.30 (s, 3H), 1.03 (s, 3H), 1.01 (s, 3H), 0.98 (s, 3H), 0.83 (d, *J* = 2.0 Hz, 3H), 0.80 (s, 3H), 0.78 (d, *J* = 7.4 Hz, 3H). ^13^C-NMR (125 MHz, C_5_D_5_N) δ 179.9, 170.1, 144.6, 122.2, 99.8, 95.3, 84.0, 81.0, 80.8, 78.7, 78.6, 77.5, 76.3, 74.1, 72.6, 69.0, 65.3, 64.3, 55.3, 54.5, 47.7, 46.5, 46.3, 42.0, 41.8, 39.5, 38.0, 37.7, 37.0, 34.0, 33.1, 33.0, 32.8, 30.6, 28.1, 27.9, 26.0, 23.7, 23.6, 23.5, 23.5, 19.1, 17.2, 15.2, 13.5. HRMS (ESI) *m*/*z* for C_39_H_64_NO_10_ ([M+H]^+^) 706.4520, calc. 706.4525.

*(3S)-O-(N-Methoxy-N-β-(2-deoxy-d-galactosyl)glycyl) oleanolic acid* (**4f**) 

White solid (29.8 mg, 43%). ^1^H-NMR (500 MHz, C_5_D_5_N) δ 5.49 (s, 1H), 4.79 (dd, *J* = 12.2, 5.2 Hz, 1H), 4.76 (d, *J* = 10.9 Hz, 1H), 4.52–4.01 (m, 6H), 3.99–3.90 (m, 1H), 3.85 (s, 3H), 3.33 (dd, *J* = 13.7, 3.7 Hz, 1H), 1.30 (s, 20H), 1.04 (s, 3H), 1.01 (s, 3H), 0.98 (s, 3H), 0.90 (s, 3H), 0.85 (s, 6H). ^13^C-NMR (125 MHz, C_5_D_5_N) δ 180.0, 170.1, 144.7, 122.2, 91.2, 80.9, 78.8, 70.2, 68.6, 62.5, 62.1, 55.3, 55.1, 47.7, 46.5, 46.3, 42.0, 41.8, 39.5, 38.0, 37.8, 37.0, 34.0, 33.1, 33.1, 33.0, 32.83, 30.18, 28.1, 27.9, 26.0, 23.7, 23.6, 23.5, 22.7, 18.3, 17.2, 16.8, 15.2. HRMS (ESI) *m*/*z* for C_39_H_64_NO_9_ ([M+H]^+^) 690.4581, calc. 690.4576.

*(3S)-O-(N-Methoxy-N-d-mannosylglycyl) oleanolic acid* (**4g**) 

White solid (17.2 mg, 24%). ^1^H-NMR (500 MHz, C_5_D_5_N) δ 5.50 (d, *J* = 12.8 Hz, 1H), 5.13 (dd, *J* = 38.5, 21.1 Hz, 2H), 4.93 (d, *J* = 1.7 Hz, 0.5H, αH1′), 4.82–4.54 (m, 3H), 4.53–4.28 (m, 3H), 4.25–4.18 (m, 0.5H), 3.96 (s, 3H), 3.79 (s, 1H), 3.44–3.25 (m, 1H), 1.03 (s, 3H), 1.00 (d, *J* = 1.6 Hz, 3H), 0.98 (s, 3H), 0.84 (d, *J* = 7.0 Hz, 3H), 0.82 (d, *J* = 2.8 Hz, 3H), 0.78 (s, 3H). ^13^C-NMR (125 MHz, C_5_D_5_N) δ 180.0, 170.1, 144.6, 122.2, 93.7, 92.1, 81.0, 81.0, 80.5, 77.9, 76.2, 74.1, 73.2, 72.2, 68.8, 65.3, 64.3, 55.6, 55.3, 55.3, 47.7, 46.5, 46.3, 42.0, 41.8, 39.5, 38.0, 37.7, 34.0, 34.0, 33.1, 33.0, 32.8, 30.6, 28.1, 27.9, 26.0, 23.8, 23.6, 23.5, 23.5, 19.1, 17.2, 15.2, 13.5. HRMS (ESI) *m*/*z* for C_39_H_64_NO_10_ ([M+H]^+^) 706.4528, calc. 706.4525.

*(3S)-O-(N-Methoxy-N-α-d-arabinosylglycyl) oleanolic acid* (**4h**) 

White solid (35.5 mg, 53%). ^1^H-NMR (500 MHz, C_5_D_5_N) δ 5.49 (s, 1H), 5.33 (d, *J* = 5.4 Hz, 1H), 4.81 (dd, *J* = 11.6, 4.7 Hz, 2H), 4.70–4.64 (m, 1H), 4.46–4.09 (m, 4H), 4.03–3.94 (m, 1H), 3.92 (s, 3H), 3.37–3.27 (m, 1H), 1.30 (d, *J* = 1.9 Hz, 3H), 1.03 (s, 3H), 1.01 (s, 3H), 0.98 (s, 3H), 0.93 (d, *J* = 4.0 Hz, 3H), 0.89 (s, 3H), 0.84 (s, 3H). ^13^C-NMR (125 MHz, C_5_D_5_N) δ 180.5, 170.5, 145.2, 122.8, 100.3, 85.7, 81.6, 79.4, 77.7, 63.1, 62.7, 55.9, 48.3, 47.0, 46.8, 42.5, 42.4, 40.1, 38.6, 38.4, 37.5, 34.6, 33.6, 33.6, 33.4, 31.9, 31.3, 30.7, 28.7, 28.5, 26.6, 24.3, 24.1, 24.1, 18.8, 17.7, 17.4, 15.7. HRMS (ESI) *m*/*z* for C_38_H_62_NO_9_ ([M+H]^+^) 676.4413, calc. 676.4419.

*(3S)-O-(N-Methoxy-N-l-arabinosylglycyl) oleanolic acid* (**4i**) 

White solid (13.7 mg, 20%). ^1^H-NMR (500 MHz, C_5_D_5_N) δ 5.50 (s, 1H), 5.32 (d, J = 5.4 Hz, 0.5H, αH1′), 4.86–4.72 (m, 2H), 4.70–4.66 (m, 0.5H), 4.64 (d, J = 9.0 Hz, 0.5H, βH1′), 4.52 (t, J = 8.9 Hz, 0.5H), 4.42–4.26 (m, 2H), 4.19 (ddd, J = 29.1, 11.0, 5.8 Hz, 2H), 3.96 (d, J = 32.9 Hz, 3H), 3.33 (dd, J = 13.8, 3.7 Hz, 1H), 1.30 (s, 3H), 1.04 (s, 3H), 1.01 (s, 3H), 0.98 (s, 3H), 0.88 (s, 3H), 0.86 (d, J = 5.8 Hz, 3H), 0.83 (d, J = 4.6 Hz, 3H). ^13^C-NMR (125 MHz, C_5_D_5_N) δ 179.9, 170.2, 144.7, 122.2, 99.8, 95.5, 85.1, 81.0, 80.9, 78.9, 77.2, 75.6, 69.8, 69.1, 62.4, 62.2, 61.7, 55.6, 55.3, 47.7, 46.5, 46.3, 42.0, 41.8, 39.5, 37.8, 37.7, 37.0, 34.0, 33.1, 33.0, 32.8, 30.8, 28.1, 27.9, 26.0, 23.7, 23.6, 23.5, 18.3, 17.2, 16.8, 15.2. HRMS (ESI) *m*/*z* for C_38_H_62_NO_9_ ([M+H]^+^) 676.4411, calc. 676.4419.

*(3S)-O-(N-Methoxy-N-d-fucosylglycyl) oleanolic acid* (**4j**) 

White solid (19.4 mg, 28%). ^1^H-NMR (500 MHz, C_5_D_5_N) δ 5.49 (s, 1H), 5.29 (d, *J* = 5.3 Hz, 0.67H, αH1′), 4.70 (d, *J* = 9.1 Hz, 0.33H, βH1′), 4.53–4.09 (m, 3H), 3.99 (s, 3H), 3.93 (s, 3H), 3.85 (dd, *J* = 12.7, 6.1 Hz, 0.5H), 3.70–3.60 (m, 0.5H), 3.32 (d, *J* = 13.1 Hz, 1H), 1.30 (s, 3H), 1.03 (s, 3H), 1.01 (s, 3H), 0.98 (s, 3H), 0.92 (d, *J* = 9.7 Hz, 3H), 0.87 (s, 3H), 0.84 (s, 3H). ^13^C-NMR (125 MHz, C_5_D_5_N) δ 180.5, 170.5, 145.2, 122.7, 100.2, 95.6, 88.0, 81.6, 81.5, 79.6, 78.4, 76.9, 73.6, 68.0, 62.8, 62.2, 56.0, 55.9, 52.3, 48.3, 47.0, 46.8, 42.5, 42.3, 40.1, 38.6, 38.3, 37.5, 34.6, 33.6, 33.6, 33.4, 31.3, 28.7, 28.5, 26.6, 24.3, 24.1, 24.1, 18.8, 17.7, 17.4, 17.3, 15.7, 14.6. HRMS (ESI) m/z for C_39_H_64_NO_9_ ([M+H]^+^) 690.4585, calc. 690.4576.

*(3S)-O-(N-Methoxy-N-l-fucosylglycyl) oleanolic acid* (**4k**) 

White solid (17.3 mg, 25%). ^1^H-NMR (500 MHz, C_5_D_5_N) δ 5.49 (s, 1H), 5.31 (d, *J* = 5.3 Hz, 0.67H, αH1′), 4.72 (d, *J* = 9.0 Hz, 0.33H, βH1′), 4.49–4.04 (m, 3H), 3.99 (s, 3H), 3.93 (s, 3H), 3.86 (q, *J* = 6.4 Hz, 0.5H), 3.64 (dd, *J* = 9.3, 5.1 Hz, 0.5H), 3.32 (d, *J* = 13.6 Hz, 1H), 1.30 (s, 3H), 1.03 (s, 3H), 1.01 (s, 3H), 0.98 (s, 3H), 0.94–0.91 (m, 3H), 0.88 (s, 3H), 0.84 (s, 3H). ^13^C-NMR (125 MHz, C_5_D_5_N) δ 180.0, 170.0, 144.7, 122.2, 99.6, 95.0, 87.5, 81.0, 80.8, 79.0, 77.8, 76.3, 73.0, 67.4, 62.2, 61.6, 56.1, 55.3, 47.7, 46.5, 46.3, 42.0, 41.8, 39.5, 38.0, 37.8, 37.0, 34.0, 33.1, 33.0, 32.8, 30.8, 28.1, 27.9, 26.0, 23.7, 23.6, 23.5, 22.7, 18.3, 17.2, 16.8, 16.7, 15.2, 14.1. HRMS (ESI) m/z for C_39_H_62_NO_9_ ([M+H]^−^) 688.4434, calc. 688.4430.

*(3S)-O-(N-Methoxy-N-β-d-xylosylglycyl) oleanolic acid* (**4l**) 

White solid (15.0 mg, 22%). ^1^H-NMR (500 MHz, C_5_D_5_N) δ 5.49 (s, 1H), 4.70 (d, *J* = 8.4 Hz, 1H), 4.53–4.29 (m, 2H), 4.27–4.12 (m, 2H), 4.04 (s, 3H), 3.77–3.58 (m, 1H), 3.32 (d, *J* = 13.3 Hz, 1H), 1.30 (s, 3H), 1.03 (s, 3H), 1.00 (s, 3H), 0.98 (s, 3H), 0.91 (s, 3H), 0.89 (s, 3H), 0.83 (s, 3H). ^13^C-NMR (125 MHz, C_5_D_5_N) δ 180.7, 170.7, 145.2, 123.4, 96.2, 81.6, 80.3, 72.0, 71.3, 69.6, 62.7, 55.9, 55.6, 48.3, 47.0, 46.9, 42.5, 42.4, 40.1, 38.6, 38.4, 37.5, 34.6, 33.7, 33.6, 33.4, 30.8, 28.7, 28.5, 26.6, 24.3, 24.2, 24.1, 23.3, 18.8, 17.7, 17.3, 15.7. HRMS (ESI) m/z for C_38_H_62_NO_9_ ([M+H]^+^) 676.4426, calc. 676.4419.

*(3S)-O-(N-Methoxy-N-β-l-xylosylglycyl) oleanolic acid* (**4m**) 

White solid (8.6 mg, 13%). ^1^H-NMR (500 MHz, C_5_D_5_N) δ 5.49 (s, 1H), 4.72 (d, *J* = 8.2 Hz, 1H), 4.58–4.36 (m, 2H), 4.34–4.13 (m, 3H), 4.04 (s, 3H), 3.32 (d, *J* = 13.6 Hz, 1H), 1.30 (s, 3H), 1.03 (s, 3H), 1.00 (s, 3H), 0.98 (s, 3H), 0.94 (s, 3H), 0.90 (s, 3H), 0.84 (s, 3H). ^13^C-NMR (125 MHz, C_5_D_5_N) δ 179.8, 170.2, 144.5, 122.9, 95.6, 80.9, 79.7, 71.4, 70.7, 69.0, 62.0, 55.3, 54.8, 47.7, 46.5, 46.3, 42.0, 41.8, 39.5, 38.0, 37.8, 37.0, 34.0, 33.1, 33.0, 32.8, 30.2, 28.1, 27.9, 26.0, 23.7, 23.6, 23.5, 22.7, 18.3, 17.2, 16.8, 15.2. HRMS (ESI) m/z for C_38_H_62_NO_9_ ([M+H]^+^) 676.4430, calc. 676.4419.

*(3S)-O-(N-Methoxy-N-β-l-lyxosylglycyl) oleanolic acid* (**4n**) 

White solid (40.2 mg, 59%). ^1^H-NMR (500 MHz, C_5_D_5_N) δ 5.49 (t, *J* = 3.3 Hz, 1H), 5.18 (d, *J* = 8.2 Hz, 1H), 4.87–4.72 (m, 3H), 4.44–4.08 (m, 4H), 3.98 (s, 3H), 3.32 (dd, *J* = 13.9, 3.7 Hz, 1H), 1.30 (s, 3H), 1.03 (s, 3H), 1.01 (s, 3H), 0.98 (s, 3H), 0.91 (s, 3H), 0.88 (s, 3H), 0.84 (s, 3H). ^13^C-NMR (125 MHz, C_5_D_5_N) δ 179.9, 170.2, 144.6, 122.2, 92.2, 81.0, 72.9, 70.5, 67.6, 66.9, 61.8, 55.3, 55.1, 47.7, 46.5, 46.3, 42.0, 41.8, 39.5, 38.0, 37.8, 37.0, 34.0, 33.1, 33.0, 32.8, 30.8, 28.1, 27.9, 26.0, 23.7, 23.6, 23.6, 23.5, 18.3, 17.2, 16.8, 15.2. HRMS (ESI) m/z for C_38_H_62_NO_9_ ([M+H]^+^) 676.4417, calc. 676.4419.

*(3S)-O-(N-Methoxy-N-l-rhamnosylglycyl) oleanolic acid* (**4o**) 

White solid (16.4 mg, 24%). ^1^H-NMR (500 MHz, C_5_D_5_N) δ 5.49 (s, 1H), 5.14-5.09 (m, 0.8H), 4.99 (s, 0.2H), 4.88 (s, 0.5H), 4.85–4.76 (m, 0.8H), 4.67 (d, *J* = 2.0 Hz, 0.2H, αH1′), 4.61 (dd, *J* = 8.8, 3.3 Hz, 0.5H), 4.47–4.38 (m, 0.6H), 4.38–4.24 (m, 1H), 4.20–4.08 (m, 0.4H), 3.97–3.77 (m, 2H), 3.74 (s, 3H), 3.31 (d, *J* = 13.2 Hz, 1H), 1.30 (s, 3H), 1.03 (s, 3H), 1.00 (d, *J* = 3.7 Hz, 3H), 0.97 (s, 3H), 0.95 (d, *J* = 6.6 Hz, 3H), 0.93 (s, 3H), 0.87 (dd, *J* = 10.8, 5.1 Hz, 3H), 0.82 (d, *J* = 10.4 Hz, 3H). ^13^C-NMR (125 MHz, C_5_D_5_N) δ 180.5, 170.4, 145.2, 122.7, 94.4, 92.4, 81.7, 81.1, 74.3, 74.0, 73.5, 72.9, 72.7, 70.4, 62.4, 61.8, 56.7, 55.9, 48.2, 47.0, 46.8, 42.5, 42.3, 40.1, 38.5, 38.3, 37.5, 34.6, 33.6, 33.6, 33.4, 31.3, 28.7, 28.6, 26.6, 24.3, 24.1, 24.1, 24.1, 19.2, 17.7, 17.4, 17.4, 15.7, 14.6. HRMS (ESI) m/z for C_39_H_64_NO_9_ ([M+H]^+^) 690.4578, calc. 690.4576.

*(3S)-O-(N-Methoxy-N-d-ribosylglycyl) oleanolic acid* (**4p**) 

White solid (42.1 mg, 62%). ^1^H-NMR (500 MHz, C_5_D_5_N) δ 5.49 (s, 1H), 5.41 (d, *J* = 2.9 Hz, 0.33H, αH1′), 5.08 (d, *J* = 8.2 Hz, 0.67H, βH1′), 4.86–4.72 (m, 2H), 4.67 (d, J = 4.9 Hz, 1H), 4.41–4.06 (m, 4H), 4.05 (s, 3H), 3.91 (d, *J* = 15.8 Hz, 1H), 3.32 (d, *J* = 12.9 Hz, 1H), 1.30 (s, 3H), 1.03 (s, 3H), 1.01 (s, 3H), 0.98 (s, 3H), 0.94 (s, 3H), 0.91 (s, 3H), 0.83 (d, *J* = 4.4 Hz, 3H). ^13^C-NMR (125 MHz, C_5_D_5_N) δ 180.3, 170.5, 145.0, 122.6, 100.7, 92.0, 85.6, 81.3, 73.2, 72.7, 72.5, 69.2, 68.8, 66.4, 63.9, 62.6, 62.5, 55.7, 48.1, 46.8, 46.6, 42.3, 42.2, 39.9, 38.4, 38.1, 37.3, 34.4, 33.4, 33.4, 33.2, 31.1, 28.5, 28.3, 26.4, 24.1, 23.9, 23.9, 23.9, 18.6, 17.5, 17.1, 15.5. HRMS (ESI) m/z for C_38_H_62_NO_9_ ([M+H]^+^) 676.4423, calc. 676.4419.

*(3S)-O-(N-Methoxy-N-l-ribosylglycyl) oleanolic acid* (**4q**) 

White solid (40.8 mg, 60%). ^1^H-NMR (500 MHz, C_5_D_5_N) δ 5.49 (s, 1H), 5.42 (d, *J* = 3.7 Hz, 0.33H, αH1′), 5.10 (d, *J* = 8.6 Hz, 0.67H, βH1′), 4.86–4.70 (m, 2H), 4.67 (dd, *J* = 9.3, 4.7 Hz, 1H), 4.35–4.16 (m, 4H), 4.04 (s, 3H), 3.95 (s, 0.33H), 3.89 (s, 0.67H), 3.32 (d, *J* = 13.6 Hz, 1H), 1.30 (d, *J* = 2.8 Hz, 3H), 1.03 (s, 3H), 1.01 (s, 3H), 0.98 (s, 3H), 0.94 (s, 3H), 0.91 (d, *J* = 6.1 Hz, 3H), 0.83 (d, *J* = 4.6 Hz, 3H). ^13^C-NMR (125 MHz, C_5_D_5_N) δ 180.5, 170.8, 145.2, 122.8, 100.9, 92.2, 85.8, 81.5, 73.4, 72.9, 72.7, 69.5, 69.0, 66.6, 64.0, 62.8, 62.7, 55.9, 48.3, 47.0, 46.8, 42.5, 42.4, 40.1, 38.6, 38.4, 37.5, 34.6, 33.6, 33.6, 33.4, 31.3, 28.7, 28.5, 26.6, 24.3, 24.1, 24.1, 24.1, 18.8, 17.7, 17.3, 15.7. HRMS (ESI) m/z for C_38_H_62_NO_9_ ([M+H]^+^) 676.4413, calc. 676.4419.

*(3S)-O-(N-Methoxy-N-(2-deoxy-d-ribosyl)glycyl) oleanolic acid* (**4r**) 

White solid (32.5 mg, 49%). ^1^H-NMR (500 MHz, C_5_D_5_N) δ 5.49 (s, 1H), 5.32 (d, *J* = 2.9 Hz, 0.8H, αH1′), 4.73–4.46 (m, 2H), 4.24–4.05 (m, 4H), 3.86 (s, 3H), 3.50 (d, *J* = 10.1 Hz, 1H), 3.35–3.29 (m, 1H), 1.30 (s, 3H), 1.03 (s, 3H), 1.01 (s, 3H), 0.98 (s, 3H), 0.93 (d, *J* = 7.5 Hz, 3H), 0.88 (d, *J* = 10.6 Hz, 3H), 0.84 (s, 3H). ^13^C-NMR (125 MHz, C_5_D_5_N) δ 180.5, 168.2, 145.2, 122.8, 106.2, 91.8, 82.3, 81.5, 72.5, 71.1, 70.8, 68.8, 68.6, 66.0, 63.3, 62.8, 62.5, 55.9, 48.3, 47.0, 46.8, 42.5, 42.4, 40.1, 38.6, 38.4, 37.5, 34.6, 33.6, 33.6, 33.4, 31.2, 28.7, 28.5, 26.6, 24.3, 24.2, 24.2, 24.1, 18.9, 17.7, 17.4, 15.8. HRMS (ESI) m/z for C_38_H_60_NO_8_ ([M+H]^−^) 658.4326, calc. 658.4324.

*28-N-Methoxyaminooleanane-β-d-glucoside* (**8a**) 

White solid (36.2 mg, 60%). ^1^H-NMR (500 MHz, C_5_D_5_N) δ 5.20 (d, *J* = 3.0 Hz, 1H), 4.61 (d, *J* = 7.9 Hz, 1H), 4.48 (dd, *J* = 11.8, 2.6 Hz, 1H), 4.36 (dd, *J* = 11.8, 4.9 Hz, 1H), 4.27–4.18 (m, 3H), 3.93–3.88 (m, 1H), 3.80 (s, 3H), 3.46–3.36 (m, 2H), 3.10 (d, *J* = 15.3 Hz, 1H), 2.27–2.09 (m, 1H), 1.21 (s, 3H), 1.20 (s, 3H), 1.05 (s, 3H), 1.02 (s, 3H), 0.89 (d, *J* = 1.3 Hz, 6H), 0.88 (s, 3H). ^13^C-NMR (125 MHz, C_5_D_5_N) δ 145.7, 123.7, 97.6, 81.1, 80.8, 78.8, 72.6, 72.5, 63.7, 56.5, 48.8, 48.0, 45.6, 42.6, 41.0, 40.2, 39.9, 38.0, 37.1, 35.6, 34.2, 33.7, 33.6, 32.3, 31.8, 31.1, 30.7, 29.5, 28.9, 27.2, 26.9, 24.8, 24.7, 19.6, 18.1, 17.3, 16.5. HRMS (ESI) *m*/*z* for C_37_H_64_NO_7_ ([M+H]^+^) 634.4676, calc. 634.4677.

*28-N-Methoxyaminooleanane-β-l-glucoside* (**8b**) 

White solid (32.2 mg, 51%). ^1^H-NMR (500 MHz, C_5_D_5_N) δ 5.19 (d, *J* = 3.0 Hz, 1H), 4.60 (d, *J* = 8.0 Hz, 1H), 4.48 (dd, *J* = 11.8, 2.5 Hz, 1H), 4.37 (dd, *J* = 11.8, 4.9 Hz, 1H), 4.30–4.22 (m, 3H), 3.86 (s, 1H), 3.81 (s, 3H), 3.44–3.38 (m, 2H), 3.22 (d, *J* = 14.9 Hz, 1H), 2.26–2.18 (m, 1H), 1.23 (s, 3H), 1.20 (s, 3H), 1.09 (s, 3H), 1.02 (s, 3H), 0.95 (s, 3H), 0.90 (s, 3H), 0.86 (s, 3H). ^13^C-NMR (125 MHz, C_5_D_5_N) δ 145.1, 123.0, 97.2, 80.5, 80.3, 78.2, 72.2, 71.9, 63.0, 55.9, 48.1, 47.4, 44.5, 42.0, 40.4, 39.5, 39.2, 37.3, 36.7, 35.0, 33.5, 33.2, 33.1, 31.6, 31.2, 30.5, 30.1, 28.9, 28.2, 26.4, 26.1, 24.0, 23.9, 18.9, 17.7, 16.7, 15.9. HRMS (ESI) *m*/*z* for C_37_H_64_NO_7_ ([M+H]^+^) 634.4673, calc. 634.4677.

*28-N-Methoxyaminooleanane-β-2-deoxy-d-glucoside* (**8c**) 

White solid (33.9 mg, 53%). ^1^H-NMR (500 MHz, C_5_D_5_N) δ 5.21 (t, *J* = 3.2 Hz, 1H), 4.72 (d, *J* = 10.5 Hz, 1H), 4.50 (dd, *J* = 11.6, 2.8 Hz, 1H), 4.39 (dd, *J* = 11.6, 4.9 Hz, 1H), 4.30–4.23 (m, 1H), 4.06 (t, *J* = 9.0 Hz, 1H), 3.86–3.80 (m, 1H), 3.66 (s, 3H), 3.44 (dd, *J* = 10.9, 4.8 Hz, 1H), 3.34 (d, *J* = 15.1 Hz, 1H), 2.96 (d, *J* = 15.2 Hz, 1H), 2.60–2.47 (m, 1H), 1.26 (s, 3H), 1.24 (s, 3H), 1.08 (s, 3H), 1.06 (s, 3H), 0.95 (s, 3H), 0.94 (s, 3H), 0.91 (s, 3H). ^13^C-NMR (125 MHz, C_5_D_5_N) δ 145.0, 123.2, 92.8, 80.5, 78.2, 73.9, 73.6, 63.3, 55.8, 48.2, 47.3, 45.0, 41.9, 40.4, 39.5, 39.2, 37.7, 37.3, 36.3, 34.9, 33.5, 33.0, 31.6, 31.1, 30.5, 30.1, 28.9, 28.2, 26.5, 26.1, 24.1, 24.0, 23.9, 18.9, 17.4, 16.6, 15.9. HRMS (ESI) *m*/*z* for C_37_H_64_NO_6_ ([M+H]^+^) 618.4730, calc. 618.4728.

*28-N-Methoxyaminooleanane-β-3-O-methyl-d-glucoside* (**8d**) 

White solid (25.2 mg, 39%). ^1^H-NMR (500 MHz, C_5_D_5_N) δ 5.27 (s, 1H), 4.63 (d, *J* = 8.9 Hz, 1H), 4.50 (dd, *J* = 11.8, 2.5 Hz, 1H), 4.39 (dd, *J* = 11.8, 4.8 Hz, 1H), 4.23 (q, *J* = 9.1 Hz, 2H), 3.95 (s, 3H), 3.85–3.77 (m, 4H), 3.50–3.41 (m, 2H), 3.13 (d, *J* = 15.3 Hz, 1H), 2.32–2.18 (m, 1H), 1.27 (s, 3H), 1.27 (s, 3H), 1.11 (s, 3H), 1.09 (s, 3H), 0.96 (s, 3H), 0.95 (s, 3H), 0.95 (s, 3H). ^13^C-NMR (125 MHz, C_5_D_5_N) δ 144.9, 122.9, 96.6, 90.0, 80.1, 78.1, 71.2, 70.8, 62.6, 60.9, 55.7, 48.0, 47.2, 44.7, 41.8, 40.2, 39.4, 39.1, 37.2, 36.3, 34.8, 33.4, 32.8, 32.8, 31.5, 31.0, 30.3, 28.7, 28.1, 26.4, 26.1, 24.1, 23.9, 23.8, 18.8, 17.3, 16.5, 15.7. HRMS (ESI) *m*/*z* for C_38_H_66_NO_7_ ([M+H]^+^) 648.4825, calc. 648.4834.

*28-N-Methoxyaminooleanane-d-galactoside* (**8e**)

White solid (35.8 mg, 56%). ^1^H-NMR (500 MHz, C_5_D_5_N) δ 5.23 (t, *J* = 5.4 Hz, 1H), 5.11 (d, *J* = 6.2 Hz, 0.2H, βH1′), 4.67 (d, *J* = 2.8 Hz, 0.8H, αH1′), 4.62 (s, 1H), 4.56–4.46 (m, 1H), 4.43–4.33 (m, 2H), 4.24 (dt, *J* = 9.5, 4.7 Hz, 1H), 4.10 (t, *J* = 6.3 Hz, 1H), 3.83–3.78 (m, 3H), 3.70–3.60 (m, 1H), 3.45 (dd, *J* = 10.9, 5.1 Hz, 1H), 3.11 (dd, *J* = 15.2, 8.0 Hz, 1H), 1.28 (s, 3H), 1.25 (s, 3H), 1.14 (d, *J* = 6.5 Hz, 3H), 1.06 (s, 3H), 0.94 (s, 3H), 0.92 (s, 3H), 0.86 (s, 3H). ^13^C-NMR (125 MHz, C_5_D_5_N) δ 145.0, 122.8, 101.7, 97.1, 83.8, 78.7, 78.3, 78.2, 78.0, 76.9, 73.0, 70.2, 69.6, 64.9, 62.2, 61.6, 55.8, 48.2, 47.3, 45.4, 42.0, 40.4, 39.5, 39.2, 37.3, 36.5, 34.8, 33.5, 33.3, 33.0, 31.1, 30.5, 30.0, 28.8, 28.2, 26.5, 26.5, 26.2, 24.0, 18.9, 17.4, 16.6, 15.8. HRMS (ESI) *m*/*z* for C_37_H_64_NO_7_ ([M+H]^+^) 634.4676, calc. 634.4677.

*28-N-Methoxyaminooleanane-2-deoxy-d-galactoside* (**8f**)

White solid (33.1 mg, 54%). ^1^H-NMR (500 MHz, C_5_D_5_N) δ 5.36 (d, *J* = 6.5 Hz, 0.33H, αH1′), 5.15 (t, *J* = 3.3 Hz, 1H), 4.69–4.62 (m, 1H), 4.52–4.35 (m, 3H), 4.32–4.28 (m, 0.67 H), 3.95 (t, *J* = 6.1 Hz, 1H), 3.67 (d, *J* = 8.1 Hz, 3H), 3.52–3.42 (m, 3H), 2.95 (d, *J* = 15.3 Hz, 1H), 1.28–1.25 (m, 6H), 1.12 (s, 3H), 1.07 (s, 3H), 0.95 (s, 6H), 0.88 (s, 3H). ^13^C-NMR (125 MHz, C_5_D_5_N) δ 144.8, 122.7, 98.0, 97.0, 92.8, 86.8, 86.3, 78.8, 78.0, 73.7, 73.1, 72.1, 71.8, 70.7, 68.6, 65.0, 64.7, 62.3, 55.7, 48.0, 48.0, 47.2, 45.1, 41.8, 41.8, 40.2, 39.3, 39.0, 37.2, 36.2, 34.7, 30.9, 28.7, 28.0, 26.3, 25.9, 23.8, 23.7, 23.6, 18.7, 17.3, 16.4, 15.7. HRMS (ESI) *m*/*z* for C_37_H_64_NO_6_ ([M+H]^+^) 618.4739, calc. 618.4728.

*28-N-Methoxyaminooleanane-d-mannoside* (**8g**)

White solid (33.5 mg, 52%). ^1^H-NMR (500 MHz, C_5_D_5_N) δ 5.24 (t, *J* = 5.4 Hz, 1H), 5.00 (d, *J* = 2.5 Hz, 0.5H, αH1′), 4.77–4.70 (m, 0.5H), 4.67 (dt, *J* = 8.3, 4.0 Hz, 1H), 4.59–4.43 (m, 2H), 4.38–4.17 (m, 1H), 3.99–3.57 (m, 5H), 3.54–3.32 (m, 2H), 3.03 (dd, *J* = 20.5, 14.9 Hz, 1H), 1.28 (d, *J* = 3.1 Hz, 3H), 1.27 (d, *J* = 2.8 Hz, 3H), 1.07 (t, *J* = 3.1 Hz, 3H), 0.98 (t, *J* = 3.5 Hz, 3H), 0.95 (s, 3H), 0.93 (d, *J* = 2.8 Hz, 3H), 0.89 (d, *J* = 11.2 Hz, 3H). ^13^C-NMR (125 MHz, C_5_D_5_N) δ 145.0, 122.9, 102.6, 96.1, 93.8, 82.0, 81.3, 78.2, 77.1, 73.4, 72.5, 71.5, 69.8, 65.2, 63.5, 62.3, 60.6, 55.9, 48.2, 47.3, 45.2, 42.0, 40.3, 39.5, 39.2, 37.3, 36.6, 35.0, 33.5, 31.6, 31.1, 30.5, 28.9, 28.2, 26.6, 26.4, 24.1, 24.0, 23.9, 18.9, 17.5, 16.6, 15.9. HRMS (ESI) *m*/*z* for C_37_H_64_NO_7_ ([M+H]^+^) 634.4672, calc. 634.4677.

*28-N-Methoxyaminooleanane-d-arabinoside* (**8h**) 

White solid (35.2 mg, 58%). ^1^H-NMR (500 MHz, C_5_D_5_N) δ 5.25 (t, *J* = 3.1 Hz, 1H), 5.18 (d, *J* = 5.5 Hz, 0.67H, αH1′), 4.67–4.63 (m, 1H), 4.61–4.50 (m, 1H), 4.46–4.34 (m, 2H), 4.29–4.21 (m, 1H), 4.16 (dd, *J* = 8.9, 3.4 Hz, 0.33H), 3.89–3.78 (m, 3H), 3.49–3.42 (m, 2H), 3.34 (dd, *J* = 50.9, 15.5 Hz, 1H), 2.28 (dd, *J* = 13.6, 3.8 Hz, 0.33H), 1.30 (s, 3H), 1.26 (d, *J* = 1.8 Hz, 3H), 1.08 (d, *J* = 2.8 Hz, 3H), 1.06 (s, 3H), 0.95 (d, *J* = 3.4 Hz, 3H), 0.93 (s, 3H), 0.90 (d, *J* = 5.7 Hz, 3H). ^13^C-NMR (125 MHz, C_5_D_5_N) δ 144.8, 122.7, 102.0, 97.6, 84.3, 78.0, 77.7, 76.3, 70.2, 69.5, 69.4, 62.7, 61.9, 55.7, 48.0, 47.2, 45.1, 41.9, 40.2, 39.3, 39.1, 37.2, 36.5, 34.8, 33.3, 33.1, 33.0, 31.4, 30.9, 30.3, 29.9, 28.7, 28.1, 26.3, 26.0, 23.9, 23.7, 18.7, 17.5, 16.5, 15.7. HRMS (ESI) m/z for C_36_H_62_NO_6_ ([M+H]^+^) 604.4581, calc. 604.4572.

*28-N-Methoxyaminooleanane-l-arabinoside* (**8i**) 

White solid (37.3 mg, 62%). ^1^H-NMR (500 MHz, C_5_D_5_N) δ 5.18 (d, *J* = 5.9 Hz, 0.5H, αH1′), 5.14 (t, *J* = 3.2 Hz, 2H), 4.63–4.59 (m, 0.5H), 4.53–4.41 (m, 2H), 4.31–4.11 (m, 3H), 3.73 (d, *J* = 6.3 Hz, 3H), 3.42 (dd, *J* = 10.9, 4.9 Hz, 1H), 3.01 (d, *J* = 15.5 Hz, 1H), 2.17 (d, *J* = 10.6 Hz, 3H), 1.25 (s, 3H), 1.21 (s, 3H), 1.11 (s, 3H), 1.02 (s, 3H), 0.94 (s, 3H), 0.88 (s, 3H), 0.78 (s, 3H). ^13^C-NMR (125 MHz, C_5_D_5_N) δ 144.9, 122.6, 101.4, 97.0, 84.6, 78.1, 76.2, 70.2, 69.6, 69.5, 55.8, 55.7, 48.1, 48.1, 47.1, 45.6, 41.9, 41.9, 40.3, 40.2, 39.4, 39.1, 37.3, 36.4, 33.4, 33.0, 31.5, 31.0, 30.4, 30.0, 28.8, 28.1, 26.4, 26.1, 23.9, 23.9, 23.5, 18.8, 17.4, 16.5, 15.8. HRMS (ESI) *m*/*z* for C_36_H_62_NO_6_ ([M+H]+) 604.4576, calc. 604.4572.

*28-N-Methoxyaminooleanane-d-fucoside* (**8j**)

White solid (35.2 mg, 57%). ^1^H-NMR (500 MHz, C_5_D_5_N) δ 5.15 (s, 1H), 4.51 (dt, *J* = 17.5, 8.7 Hz, 2H), 4.18 (dd, *J* = 8.7, 3.4 Hz, 1H), 4.09 (d, *J* = 2.8 Hz, 1H), 3.88 (q, *J* = 6.0 Hz, 1H), 3.78 (s, 3H), 3.66 (t, *J* = 9.8 Hz, 1H), 3.47 (dd, *J* = 11.0, 4.9 Hz, 1H), 3.10–3.04 (m, 1H), 1.29 (s, 3H), 1.27 (s, 3H), 1.19 (s, 3H), 1.08 (s, 3H), 0.99 (s, 3H), 0.93 (s, 3H), 0.86 (s, 3H). ^13^C-NMR (125 MHz, C_5_D_5_N) δ 145.5, 123.3, 97.2, 87.6, 78.7, 77.4, 74.0, 73.5, 69.7, 56.4, 48.7, 47.8, 46.1, 42.5, 40.9, 40.0, 39.7, 37.9, 37.0, 35.3, 34.0, 33.9, 33.6, 32.1, 31.6, 31.0, 30.6, 29.4, 28.7, 27.0, 26.7, 24.5, 19.4, 18.1, 18.0, 17.1, 16.4. HRMS (ESI) *m*/*z* for C_37_H_64_NO_6_ ([M+H]+)618.4733, calc. 618.4728.

*28-N-Methoxyaminooleanane-β-l-fucoside* (**8k**) 

White solid (38.9 mg, 63%). ^1^H-NMR (500 MHz, C_5_D_5_N) δ 5.19 (d, *J* = 3.2 Hz, 1H), 4.49 (t, *J* = 8.8 Hz, 1H), 4.44 (d, *J* = 8.7 Hz, 1H), 4.10 (dd, *J* = 8.8, 3.4 Hz, 1H), 4.04 (d, *J* = 2.9 Hz, 1H), 3.80 (d, *J* = 6.9 Hz, 1H), 3.74 (s, 3H), 3.42 (dd, *J* = 10.9, 4.9 Hz, 1H), 3.35 (dd, *J* = 14.9, 3.9 Hz, 1H), 3.26 (d, *J* = 15.0 Hz, 1H), 2.26 (dd, *J* = 13.4, 3.9 Hz, 1H), 1.25 (s, 3H), 1.22 (s, 3H), 1.14 (s, 3H), 1.02 (d, *J* = 2.3 Hz, 3H), 0.97 (s, 3H), 0.91 (s, 6H). ^13^C-NMR (125 MHz, C_5_D_5_N) δ 145.0, 122.7, 96.9, 86.6, 78.0, 76.9, 73.3, 72.8, 68.9, 55.7, 48.0, 47.3, 44.3, 41.9, 40.2, 39.3, 39.1, 37.2, 37.2, 36.4, 34.8, 33.3, 33.0, 32.8, 31.0, 28.7, 28.0, 26.2, 25.9, 23.9, 23.8, 20.5, 18.7, 17.4, 17.4, 16.5, 15.7. HRMS (ESI) *m*/*z* for C_37_H_64_NO_6_ ([M+H]^+^) 618.4732, calc. 618.4728.

*28-N-Methoxyaminooleanane-β-d-xyloside* (**8l**) 

White solid (38.2 mg, 63%). ^1^H-NMR (500 MHz, C_5_D_5_N) δ 5.26 (t, *J* = 3.3 Hz, 1H), 4.57 (d, *J* = 6.7 Hz, 1H), 4.40 (dd, *J* = 10.8, 4.5 Hz, 1H), 4.19 (d, *J* = 13.9 Hz, 3H), 3.80 (s, 3H), 3.69 (t, *J* = 10.4 Hz, 1H), 3.52 (d, *J* = 15.3 Hz, 1H), 3.41 (t, *J* = 14.6 Hz, 1H), 3.03 (d, *J* = 15.4 Hz, 1H), 2.25–2.14 (m, 1H), 1.26 (s, 3H), 1.23 (s, 3H), 1.10 (s, 3H), 1.04 (s, 3H), 0.92 (s, 3H), 0.92 (s, 3H), 0.89 (s, 3H). ^13^C-NMR (125 MHz, C_5_D_5_N) δ 144.9, 122.7, 97.1, 80.1, 78.0, 71.7, 70.9, 69.2, 55.7, 48.0, 47.0, 45.3, 41.8, 40.2, 39.3, 39.0, 37.2, 36.2, 34.7, 33.3, 33.1, 32.8, 31.4, 31.0, 30.3, 28.7, 28.0, 26.3, 26.1, 23.8, 23.8, 23.6, 18.7, 17.2, 16.4, 15.7. HRMS (ESI) *m*/*z* for C_36_H_62_NO_6_ ([M+H]^+^) 604.4572, calc. 604.4572.

*28-N-Methoxyaminooleanane-β-l-xyloside* (**8m**) 

White solid (37.2 mg, 62%). ^1^H-NMR (500 MHz, C_5_D_5_N) δ 5.30 (t, *J* = 3.2 Hz, 1H), 4.55 (d, *J* = 8.7 Hz, 1H), 4.43 (dd, *J* = 11.1, 5.2 Hz, 1H), 4.28–4.18 (m, 3H), 3.85 (s, 3H), 3.70 (t, *J* = 10.7 Hz, 1H), 3.49–3.43 (m, 1H), 3.34 (d, *J* = 5.8 Hz, 2H), 2.29 (dd, *J* = 13.3, 3.8 Hz, 1H), 1.29 (s, 3H), 1.25 (s, 3H), 1.17 (s, 3H), 1.06 (s, 3H), 0.99 (s, 3H), 0.95 (s, 3H), 0.92 (s, 3H). ^13^C-NMR (125 MHz, C_5_D_5_N) δ 145.0, 122.8, 97.8, 80.2, 78.1, 71.7, 71.1, 69.3, 55.7, 49.6, 48.0, 47.2, 44.7, 42.0, 40.3, 39.4, 39.1, 37.2, 36.5, 34.8, 33.4, 33.1, 33.0, 31.5, 31.0, 30.3, 28.7, 28.1, 26.3, 26.0, 23.9, 23.8, 18.8, 17.5, 16.5, 15.7. HRMS (ESI) *m*/*z* for C_36_H_62_NO_6_ ([M+H]^+^) 604.4578, calc. 604.4572.

*28-N-Methoxyaminooleanane-l-lyxoside* (**8n**)

White solid (34.2 mg, 57%). ^1^H-NMR (500 MHz, C_5_D_5_N) δ 5.88 (d, *J* = 3.5 Hz, 0.2H, αH1′), 5.22 (d, *J* = 3.2 Hz, 1H), 4.81 (d, *J* = 3.3 Hz, 1H), 4.78–4.72 (m, 1H), 4.72–4.65 (m, 0.8H), 4.49–4.42 (m, 1H), 4.35 (dd, *J* = 11.2, 4.2 Hz, 1H), 4.23 (dd, *J* = 18.4, 8.8 Hz, 1H), 3.97–3.78 (m, 3H), 3.60–3.43 (m, 2H), 3.09 (dd, *J* = 16.5, 15.1 Hz, 1H), 1.29–1.27 (m, 3H), 1.25 (d, *J* = 11.2 Hz, 3H), 1.16 (d, *J* = 5.4 Hz, 3H), 1.09–1.06 (m, 3H), 0.98 (d, *J* = 7.0 Hz, 3H), 0.95 (d, *J* = 3.8 Hz, 3H), 0.91 (d, *J* = 3.2 Hz, 3H). ^13^C-NMR (125 MHz, C_5_D_5_N) δ 145.56, 123.23, 102.50, 96.98, 83.26, 78.68, 73.85, 73.61, 73.41, 71.79, 69.99, 68.58, 67.66, 56.35, 50.22, 48.67, 47.71, 46.17, 42.49, 40.87, 39.97, 39.69, 37.83, 36.97, 35.39, 34.02, 33.98, 33.79, 33.52, 31.59, 30.94, 29.33, 28.70, 26.97, 26.73, 24.49, 24.43, 19.39, 17.92, 17.11, 16.34. HRMS (ESI) *m*/*z* for C_36_H_62_NO_6_ ([M+H]^+^) 604.4575, calc. 604.4572.

*28-N-Methoxyaminooleanane-l-rhamnoside* (**8o**)

White solid (34.7 mg, 56%). ^1^H-NMR (500 MHz, C_5_D_5_N) δ 5.33 (t, *J* = 3.3 Hz, 1H), 5.28 (d, *J* = 6.1 Hz, 0.2H, βH1′), 4.69 (d, *J* = 2.9 Hz, 0.8H, αH1′), 4.34–4.29 (m, 1H), 4.19 (t, *J* = 9.1 Hz, 1H), 4.07 (dd, *J* = 9.2, 3.0 Hz, 1H), 3.84 (d, *J* = 7.8 Hz, 3H), 3.76 (dq, *J* = 9.3, 6.1 Hz, 1H), 3.51–3.39 (m, 4H), 3.12 (d, *J* = 14.6 Hz, 1H), 1.18 (s, 3H), 1.08 (d, *J* = 3.1 Hz, 3H), 1.07 (s, 3H), 1.01 (s, 3H), 0.99 (s, 3H), 0.98 (s, 6H). ^13^C-NMR (125 MHz, C_5_D_5_N) δ 145.8, 123.2, 102.2, 95.3, 86.4, 78.7, 77.3, 76.9, 74.6, 72.5, 63.8, 62.3, 61.6, 56.4, 48.7, 48.0, 45.2, 42.5, 40.9, 40.0, 40.0, 39.7, 37.9, 37.1, 34.0, 32.1, 31.6, 31.6, 31.0, 30.6, 29.4, 29.3, 28.7, 27.0, 26.6, 24.5, 24.4, 19.4, 19.3, 18.3, 17.1, 17.1, 16.4. HRMS (ESI) *m*/*z* for C_37_H_64_NO_6_ ([M+H]^+^) 618.4733, calc. 618.4728.

*28-N-Methoxyaminooleanane-d-riboside* (**8p**) 

White solid (34.9 mg, 58%). ^1^H-NMR (500 MHz, C_5_D_5_N) δ 5.29 (t, *J* = 3.0 Hz, 1H), 5.23 (d, *J* = 3.4 Hz, 0.5H, αH1′), 4.80–4.62 (m, 2H), 4.35 (dd, *J* = 11.6, 3.7 Hz, 0.5H), 4.31–4.14 (m, 3H), 3.91–3.77 (m, 3H), 3.50–3.34 (m, 2H), 3.04 (dd, *J* = 38.0, 15.1 Hz, 1H), 1.28 (d, *J* = 2.7 Hz, 3H), 1.27 (s, 3H), 1.08 (d, *J* = 2.6 Hz, 3H), 0.97 (dd, *J* = 12.2, 3.2 Hz, 6H), 0.95 (d, *J* = 4.4 Hz, 3H), 0.93 (d, *J* = 3.9 Hz, 3H). ^13^C-NMR (125 MHz, C_5_D_5_N) δ 145.3, 122.9, 103.6, 93.4, 84.7, 78.3, 73.0, 72.6, 69.3, 68.8, 66.4, 64.1, 62.2, 56.0, 55.9, 48.2, 47.3, 45.6, 42.1, 40.5, 39.6, 39.3, 37.4, 36.6, 33.6, 31.7, 31.3, 30.6, 29.0, 28.9, 28.3, 26.6, 26.5, 26.4, 24.1, 24.1, 24.0, 19.0, 17.4, 16.7, 15.9. HRMS (ESI) *m*/*z* for C_36_H_62_NO_6_ ([M+H]^+^) 604.4580, calc. 604.4572.

*28-N-Methoxyaminooleanane-l-riboside* (**8q**) 

White solid (33.2 mg, 55%). ^1^H-NMR (500 MHz, C_5_D_5_N) δ 5.24 (t, *J* = 3.2 Hz, 1H), 5.20 (d, *J* = 3.1 Hz, 0.5H, αH1′), 4.81–4.60 (m, 2H), 4.38 (dd, *J* = 11.7, 3.6 Hz, 0.5H), 4.31–4.18 (m, 3H), 3.84 (s, 3H), 3.46 (dd, *J* = 10.8, 5.1 Hz, 1H), 3.33 (dd, *J* = 14.9, 8.7 Hz, 1H), 2.98 (d, *J* = 14.8 Hz, 1H), 1.26 (s, 3H), 1.17 (s, 3H), 1.07 (s, 3H), 1.03 (s, 3H), 0.95 (s, 3H), 0.94 (d, *J* = 2.3 Hz, 3H), 0.93 (s, 3H). ^13^C-NMR (125 MHz, C_5_D_5_N) δ 145.0, 123.0, 104.3, 94.0, 84.3, 78.3, 73.1, 72.7, 69.2, 68.9, 64.0, 62.5, 55.9, 48.3, 47.3, 45.2, 44.9, 42.2, 42.1, 40.6, 40.4, 39.6, 39.3, 37.4, 37.4, 36.8, 36.7, 35.0, 33.6, 33.6, 31.2, 28.9, 28.3, 26.5, 26.5, 26.3, 24.1, 24.0, 23.8, 19.0, 17.6, 16.7, 15.9. HRMS (ESI) *m*/*z* for C_36_H_62_NO_6_ ([M+H]^+^) 604.4575, calc. 604.4572.

*28-N-Methoxyaminooleanane-2-deoxy-d-riboside* (**8r**)

White solid (33.9 mg, 58%). ^1^H-NMR (500 MHz, C_5_D_5_N) δ 5.30 (d, *J* = 3.3 Hz, 0.8H, αH1′), 5.25 (s, 1H), 4.70–4.52 (m, 1H), 4.47–4.34 (m, 1H), 4.28 (d, *J* = 10.5 Hz, 0.2H), 4.26–4.07 (m, 3H), 3.66 (d, *J* = 10.0 Hz, 3H), 3.50–3.44 (m, 2H), 1.30 (s, 3H), 1.28 (d, *J* = 3.5 Hz, 3H), 1.27 (s, 3H), 1.08 (s, 3H), 0.98 (d, *J* = 6.0 Hz, 3H), 0.97 (t, *J* = 3.0 Hz, 3H), 0.95 (s, 3H). ^13^C-NMR (125 MHz, C_5_D_5_N) δ 145.6, 123.4, 98.5, 93.5, 88.3, 78.7, 72.8, 72.1, 68.9, 68.9, 67.2, 62.6, 61.7, 60.3, 56.3, 48.6, 47.7, 47.2, 46.0, 45.6, 42.5, 40.9, 39.99, 39.72, 37.8, 36.8, 34.0, 31.6, 31.0, 29.3, 28.7, 27.0, 26.8, 26.7, 24.5, 24.4, 24.4, 19.4, 17.8, 17.1, 16.4. HRMS (ESI) *m*/*z* for C_36_H_62_NO_5_ ([M+H]^+^) 588.4616, calc. 588.4623.

*Oleanolic acid-28-O-β-d-glucopyranoside* (**1a**)

White solid (75.2 mg, 28%). ^1^H-NMR (500 MHz, C_5_D_5_N) δ 6.34 (d, *J* = 8.0 Hz, 1H), 5.46 (s, 1H), 4.48–4.03 (m, 6H), 3.44 (dd, *J* = 10.9, 5.1 Hz, 1H), 3.22 (dd, *J* = 14.1, 4.4 Hz, 1H), 1.25 (s, 3H), 1.23 (s, 3H), 1.14 (s, 3H), 1.03 (s, 3H), 0.93 (s, 3H), 0.90 (s, 3H), 0.88 (s, 3H). ^13^C-NMR (125 MHz, C_5_D_5_N) δ 176.5, 144.2, 123.0, 95.8, 79.4, 78.9, 78.1, 75.7, 74.2, 62.3, 55.8, 48.2, 47.1, 46.3, 42.2, 41.8, 39.9, 39.4, 39.0, 37.4, 34.0, 33.2, 33.2, 32.6, 31.0, 29.1, 28.8, 28.3, 28.1, 26.1, 23.9, 23.5, 18.9, 17.6, 16.6, 15.7 HRMS (ESI) *m*/*z* for C_36_H_58_O_8_Na ([M+Na]^+^) 641.4029, calc. 641.4024.

*Erythrodiol-28-O-β-d-glucopyranoside* (**1b**)

White solid (62.8 mg, 23%). ^1^H-NMR (500 MHz, C_5_D_5_N) δ 5.24 (s, 1H), 4.92 (d, *J* = 7.7 Hz, 1H), 4.62 (d, *J* = 11.7 Hz, 1H), 4.48 (dd, *J* = 11.2, 4.4 Hz, 1H), 4.35–4.28 (m, 2H), 4.10 (dd, *J* = 17.3, 8.2 Hz, 2H), 3.86 (s, 2H), 3.51–3.41 (m, 1H), 1.26 (s, 6H), 1.08 (s, 3H), 1.02 (s, 3H), 0.97 (s, 3H), 0.92 (s, 3H), 0.88 (s, 3H). ^13^C-NMR (125 MHz, C_5_D_5_N) δ 144.7, 122.8, 105.9, 78.8, 78.6, 78.1, 77.2, 75.4, 71.8, 63.0, 55.8, 48.1, 46.7, 43.2, 42.0, 40.2, 39.5, 39.2, 37.3, 37.2, 34.5, 33.5, 32.9, 32.7, 31.2, 28.8, 28.2, 28.2, 26.3, 23.9, 23.9, 22.1, 18.9, 17.1, 16.6, 15.9. HRMS (ESI) *m*/*z* for C_36_H_60_O_7_Na ([M+Na]^+^) 627.4231, calc. 627.4231.

### 3.3. Biotransformation Procedure

Cultures were grown by a two-stage procedure in 250 mL culture flasks. The culture flasks held one fifth of their volume of potato dextrose (PD) medium: peeled potatoes (400 g) were cut into pieces, boiled in water for 15 min and filtered, then glucose (60 g), KH_2_PO_4_ (6 g), MgSO_4_·7H_2_O (3 g), Vitamin B (120 mg) was added to the filtrate which was diluted with distilled water to 2 L and was adjusted to pH 7.0 with 6N HCl before being autoclaved at 121 °C for 20 min. Cultures were incubated with shaking at 28 °C on a rotary shaker at 180 rpm. One milliliter of *Bacillus subtilis* ATCC 6633 inoculum derived from 24 h-old stage I cultures was used to initiate stage II cultures, which were incubated for 24 h before adding 10 mg of the substrates in 0.5 mL of dimethyl sulfoxide. Culture controls were consisted of medium without substrates and substrate controls consisting of sterile medium as well as substrate, incubated under the same conditions but without microorganism.

### 3.4. Extraction, Isolation and Identification of Metabolites

Cultures were incubated for 96 h and extracted with equal volume of ethyl acetate for three times. The organic solvent layer was concentrated in vacuo and spotted on silica gel plates, which were developed by CH_2_Cl_2_/MeOH (9:1, *v*/*v*). The results were visualized by TLC. The metabolites were isolated by silica gel column chromatography and eluted with CH_2_Cl_2_/MeOH gradient ranging 99:1 to 90:10 (*v*/*v*).

### 3.5. Antiproliferative Activities

Five human cancer cell lines including human non-small cell lung cancer cell line (A549), human liver carcinoma cell line (HepG2), human breast adenocarcinoma cell line (MCF-7), human melanoma cell line (A375), human colon cancer cell line (HCT116) were obtained from the Cell Bank of the Chinese Academy of Science (Shanghai, China). These cell lines were cultured with standard methods in DMEM or 1640 medium containing 10% FBS (Gibco, CA, USA) and supplemented with 100 U/mL penicillin and 100 U/mL streptomycin. All compounds and 5-FU were initially dissolved in DMSO to make stock solutions and then DMEM or 1640 medium was used to dilute the stock solutions to the desired concentrations. The DMSO concentration was kept below 0.05% which was non-toxic to the cells. Cytotoxic activities were investigated using the CCK-8 assay. In brief, the exponentially growing cells were seeded on 96-well plates (6 × 10^3^ cells per well) and incubated for 24 h. Subsequently, cells were treated with designated concentrations of compounds for 72 h. Then, 10 µL of CCK-8 was added to each well, the plates were incubated at 37 °C for an additional 3 h, and then, absorbance was read at 450 nm with a reference measurement at 650 nm. Cell viability was calculated using the following formula: Relative cell viability = (OD value for the test group − blank OD)/(control OD value − blank OD value) ×100%. The half maximal inhibitory concentration (IC_50_) values were determined using GraphPad Prism 5 software (Graph Pad, La Jolla, CA, USA).

### 3.6. Hoechst 33342 Staining

HepG2 cells were plated 6-well tissue culture plates and incubated for 24 h before the treatment. Cells were treated with **8a** for 24 h before incubation with Hoechst 33342. Removed the culture medium containing compounds and fixed the cells in 4% paraformaldehyde for 30 min at room temperature. The cells were stained with 1 mL of Hoechst 33342 for 10 min and then washed twice with PBS. The stained nuclei were observed by fluorescence microscope (Olympus, Tokyo, Japan).

### 3.7. Cell Cycle Distribution Analysis

Flow cytometry was employed to determine the effect of compound **8a** on the cell cycle of HepG2 cells. We used PI to stain the DNA and RNase A to hydrolyze the phosphodiester bond between the nucleotides. Briefly, HepG2 cells were seeded into six-well plates for attaching overnight. The cells were then incubated with **8a** at concentrations of 1, 5 and 10 µM for 24 h. Cells were collected and washed twice with PBS. Cells were fixed with cold 70% ethanol at 4 °C overnight. Fixed cells were washed with PBS, and then stained with 50µg/mL propidium iodide (PI) solution containing 25µg/mL RNase A for 30 min in the dark at room temperature. Fluorescence intensity was analyzed by FACSCalibur flow cytometer (BD Biosciences, San Jose, CA, USA). The percentages of the cells distributed in different phases of the cell cycle were analyzed using ModFit LT 2.0 (Verity Software House, Topsham, ME, USA).

### 3.8. Flow Cytometry Analysis Of Apoptosis

Cell apoptosis was analyzed using the Annexin V-FITC/PI Apoptosis kit (BD Biosciences) according to the manufacturer’s protocols. Briefly, HepG2 cells were seeded into six-well plates for attaching overnight. The cells were then incubated with **8a** at concentrations of 1, 5 and 10 µM for 24 h. Cells were collected and then washed twice with cold PBS, and then stained using the annexin V-fluorescein isothiocyanate (FITC) and PI according to the manufacturer’s instructions. The stained cells were incubated for 15 min in the dark at room temperature, and the fluorescent intensity was measured using a FACSCalibur flow cytometer (BD Biosciences).

### 3.9. Statistical Analysis

Data are presented as the mean ± standard deviation (SD) from three independent experiments. Comparisons of different groups was evaluated by one-way analysis of variance (ANOVA). Statistical significance and IC_50_ values were performed using GraphPad Prism 5 software. Values of *p* < 0.05 were considered statistically significant.

## 4. Conclusions

In summary, a series of C-3 and C-28 MeON-neoglycosides of oleanolic acid were synthesized by a neoglycosylation method and their cytotoxicity on five human cancer cell lines were evaluated by the CCK-8 assay. The preliminary activity results suggested that most of neoglycosides possessed notably inhibitory effects against the tested cancer cells and exerted selective growth inhibition against HepG2 cells. Of these compounds, compound **8a** was the most potent one against HepG2 cells (IC_50_ = 2.1 µM). Further studies revealed that compound **8a** caused morphological changes, cell cycle arrest at G0/G1 phase and induced the apoptosis of HepG2 cells in a concentration-dependent manner. Hence, compound **8a** could be a promising anticancer candidate for further exploration. Collectively, our findings also suggested that neoglycosylation could be a practical tool for enrich the natural product glycodiversification.

## Figures and Tables

**Table 1:** IC_50_ (µM) ^a^ values of neoaglycone **3** and neoglycosides **4a–4r** against five human cancer cell lines.

Compound	A549	HepG2	MCF-7	A375	HCT116
**OA**	>30	>30	>30	>30	>30
**2**	>30	>30	>30	>30	>30
**3**	>30	>30	25.6 ± 0.1	>30	23.8 ± 1.6
**4a**	4.7 ± 0.9	4.3 ± 0.3	10.6 ± 0.4	11.4 ± 0.2	11.1 ± 0.1
**4b**	14.7 ± 0.3	6.5 ± 1.0	20.5 ± 0.9	24.3 ± 1.5	23.0 ± 0.4
**4c**	11.6 ± 1.9	4.2 ± 0.9	10.4 ± 0.3	11.7 ± 0.4	21.0 ± 1.7
**4d**	>30	11.6 ± 0.7	>30	>30	>30
**4e**	>30	5.2 ± 0.6	>30	23.7 ± 2.3	22.8 ± 0.2
**4f**	>30	>30	>30	>30	>30
**4g**	24.3 ± 1.2	9.5 ± 0.6	>30	15.7 ± 0.9	27.2 ± 3.5
**4h**	6.4 ± 0.8	7.3 ± 0.5	11.2 ± 0.8	9.6 ± 1.2	11.0 ± 0.1
**4i**	15.3 ± 0.7	15.5 ± 1.1	23.0 ± 1.0	18.9 ± 0.1	22.9 ± 0.2
**4j**	26.6 ± 2.0	17.7 ± 1.3	29.1 ± 2.1	24.7 ± 2.3	>30
**4k**	>30	>30	>30	28.4 ± 1.0	>30
**4l**	21.7 ± 1.0	22.4 ± 1.8	14.7 ± 1.1	11.6 ± 0.4	9.8 ± 1.9
**4m**	14.0 ± 0.8	10.5 ± 0.7	10.0 ± 0.5	9.7 ± 0.7	5.8 ± 0.3
**4n**	10.5 ± 0.3	25.0 ± 1.4	10.5 ± 0.4	8.4 ± 0.3	6.0 ± 0.9
**4o**	14.2 ± 1.2	23.7 ± 2.1	10.7 ± 0.9	11.5 ± 0.1	9.9 ± 0.3
**4p**	6.0 ± 0.4	7.3 ± 0.9	6.4 ± 0.8	6.0 ± 0.1	5.0 ± 1.2
**4q**	8.9 ± 1.7	10.2 ± 0.6	8.7 ± 0.6	10.9 ± 1.2	11.3 ± 0.2
**4r**	>30	22.3 ± 1.2	>30	>30	17.4 ± 1.6
**5-FU** ^b^	34.2 ± 2.6	29.3 ± 3.9	10.9 ± 1.2	28.5 ± 2.8	20.6 ± 1.6

a: Each value was determined in triplicate. The cells were continuously treated with compounds for 72 h. b: 5-FU was used as positive control.

**Table 2:** IC_50_ (µM) ^a^ values of neoaglycone **7** and neoglycosides **8a–8r** against five human cancer cell lines.

Compound	A549	HepG2	MCF-7	A375	HCT116
**OA**	>30	>30	>30	>30	>30
**5**	>30	>30	>30	>30	>30
**6**	>30	>30	>30	>30	>30
**7**	17.6 ± 1.8	>30	>30	>30	>30
**8a**	4.2 ± 0.6	2.1 ± 0.3	5.9 ± 0.8	10.8 ± 0.8	22.1 ± 0.1
**8b**	15.3 ± 1.2	5.2 ± 0.9	10.8 ± 1.0	14.8 ± 0.7	23.2 ± 1.0
**8c**	14.9 ± 1.8	>30	>30	13.8 ± 2.9	>30
**8d**	7.5 ± 1.0	4.1 ± 0.3	10.6 ± 1.1	26.4 ± 2.6	>30
**8e**	12.4 ± 1.3	5.1 ± 0.6	22.7 ± 2.8	14.6 ± 0.3	29.8 ± 2.7
**8f**	11.3 ± 0.8	>30	18.7 ± 0.5	13.9 ± 0.3	>30
**8g**	5.2 ± 0.9	4.5 ± 0.7	16.2 ± 1.2	13.0 ± 1.0	29.9 ± 2.6
**8h**	15.5 ± 2.3	26.7 ± 2.0	>30	27.3 ± 0.9	>30
**8i**	>30	>30	>30	>30	>30
**8j**	>30	>30	>30	>30	>30
**8k**	7.2 ± 1.5	12.1 ± 1.2	16.6 ± 0.8	17.0 ± 0.5	22.4 ± 0.3
**8l**	>30	>30	>30	>30	>30
**8m**	11.7 ± 0.9	4.5 ±0.6	8.2 ± 1.2	12.3 ± 1.6	22.9 ± 0.6
**8n**	>30	>30	>30	>30	>30
**8o**	14.6 ± 1.3	>30	15.1 ± 1.0	11.8 ± 1.5	>30
**8p**	>30	>30	>30	21.9 ± 2.9	>30
**8q**	15.2 ± 2.4	>30	>30	17.8 ± 1.7	28.4 ± 1.1
**8r**	15.3 ± 0.7	>30	16.1 ± 1.4	23.0 ± 1.6	>30
**1a**	>30	>30	>30	>30	>30
**1b**	>30	>30	>30	>30	>30
**5-FU** ^b^	34.2 ± 2.6	29.3 ± 3.9	10.9 ± 1.2	28.5 ± 2.8	20.6 ± 1.6

a: Each value was determined in triplicate. The cells were continuously treated with compounds for 72 h. b: 5-FU was used as positive control.

## Data Availability

Data sharing not applicable.

## Supplementary Materials

Table S1: ^1^H NMR anomeric proton and HRMS characterization of C-3 or C-28 MeON-neoglycosides of oleanolic acid, Figure S1: NMR spectra of neoaglycone and representative neoglycosides.

## Sample Availability

Samples of the compounds are not available from the authors.

## References

[1] Weymouth-Wilson A.C. (1997). The role of carbohydrates in biologically active natural products. Nat. Prod. Rep..

[2] Newman D.J., Cragg G.M. (2007). Natural products as sources of new drugs over the last 25 years. J. Nat. Prod..

[3] Calvaresi E.C., Hergenrother P.J. (2013). Glucose conjugation for the specific targeting and treatment of cancer. Chem. Sci..

[4] Vander Heiden M.G., Cantley L.C., Thompson C.B. (2009). Understanding the Warburg effect: The metabolic requirements of cell proliferation. Science.

[5] Cantor J.R., Sabatini D.M. (2012). Cancer cell metabolism: One hallmark, many faces. Cancer Discov..

[6] Guo N., Tong T., Ren N., Tu Y., Li B. (2018). Saponins from seeds of Genus Camellia: Phytochemistry and bioactivity. Phytochemistry.

[7] Krief S., Thoison O., Sevenet T., Wrangham R.W., Lavaud C. (2005). Triterpenoid saponin anthranilates from Albizia grandibracteata leaves ingested by primates in Uganda. J. Nat. Prod..

[8] Wang S.R., Fang W.S. (2009). Pentacyclic triterpenoids and their saponins with apoptosis-inducing activity. Curr. Top. Med. Chem..

[9] Xu Q.L., Wang J., Dong L.M., Zhang Q., Luo B., Jia Y.X., Wang H.F., Tan J.W. (2016). Two new pentacyclic triterpene saponins from the leaves of Akebia trifoliata. Molecules.

[10] Zong J., Wang R., Bao G., Ling T., Zhang L., Zhang X., Hou R. (2015). Novel triterpenoid saponins from residual seed cake of Camellia oleifera Abel. show anti-proliferative activity against tumor cells. Fitoterapia.

[11] Zhang J., Akihisa T., Kurita M., Kikuchi T., Zhu W.-F., Ye F., Dong Z.-H., Liu W.-Y., Feng F., Xu J. (2018). Melanogenesis-inhibitory and cytotoxic activities of triterpene glycoside constituents from the bark of Albizia procera. J. Nat. Prod..

[12] Langenhan J.M., Peters N.R., Guzei I.A., Hoffmann F.M., Thorson J.S. (2005). Enhancing the anticancer properties of cardiac glycosides by neoglycorandomization. Proc. Natl. Acad. Sci. USA.

[13] Nandurkar N.S., Zhang J., Ye Q., Ponomareva L.V., She Q.B., Thorson J.S. (2014). The identification of perillyl alcohol glycosides with improved antiproliferative activity. J. Med. Chem..

[14] Goff R.D., Thorson J.S. (2012). Enhancement of cyclopamine via conjugation with nonmetabolic sugars. Org. Lett..

[15] Li X.S., Ren Y.C., Bao Y.Z., Liu J., Zhang X.K., Zhang Y.W., Sun X.L., Yao X.S., Tang J.S. (2018). Synthesis of C3-Neoglycosides of digoxigenin and their anticancer activities. Eur. J. Med. Chem..

[16] Zhang J., Ponomareva L.V., Nandurkar N.S., Yuan Y., Fang L., Zhan C.G., Thorson J.S. (2015). Influence of sugar amine regiochemistry on digitoxigenin neoglycoside anticancer activity. ACS Med. Chem. Lett..

[17] Wang D.-D., Li X.-S., Bao Y.-Z., Liu J., Zhang X.-K., Yao X.-S., Sun X.-L., Tang J.-S. (2017). Synthesis of MeON-neoglycosides of digoxigenin with 6-deoxy- and 2,6-dideoxy- d -glucose derivatives and their anticancer activity. Bioorganic Med. Chem. Lett..

[18] Du Z., Li G., Ge H., Zhou X., Zhang J. (2021). Design, synthesis and biological evaluation of steroidal glycoconjugates as potential antiproliferative agents. ChemMedChem.

[19] Goff R.D., Thorson J.S. (2009). Enhancing the divergent activities of betulinic acid via neoglycosylation. Org. Lett..

[20] Li G.L., Xu H.J., Xu S.H., Wang W.W., Yu B.Y., Zhang J. (2018). Synthesis of tigogenin MeON-Neoglycosides and their antitumor activity. Fitoterapia.

[21] Peri F., Dumy P., Mutter M. (1998). Chemo- and stereoselective glycosilation of hydroxylamino derivatives: A versatile approach to glycoconjugates. Tetrahedron.

[22] Zhang J., Ponomareva L.V., Marchillo K., Zhou M., Andes D.R., Thorson J.S. (2013). Synthesis and antibacterial activity of doxycycline neoglycosides. J. Nat. Prod..

[23] Peltier-Pain P., Timmons S.C., Grandemange A., Benoit E., Thorson J.S. (2011). Warfarin glycosylation invokes a switch from anticoagulant to anticancer activity. ChemMedChem.

[24] Wang W.-W., Xu S.-H., Zhao Y.-Z., Zhang C., Zhang Y.-Y., Yu B.-Y., Zhang J. (2017). Microbial hydroxylation and glycosylation of pentacyclic triterpenes as inhibitors on tissue factor procoagulant activity. Bioorganic Med. Chem. Lett..

[25] Shen P., Wang W., Xu S., Du Z., Wang W., Yu B., Zhang J. (2020). Biotransformation of erythrodiol for new food supplements with anti-inflammatory properties. J. Agric. Food Chem..

[26] Ginsburg E., Salomon D., Sreevalsan T., Freese E. (1973). Growth inhibition and morphological changes caused by lipophilic acids in mammalian cells. Proc. Natl. Acad. Sci. USA.

[27] Kohn K.W., Jackman J., O’Connor P.M. (1994). Cell cycle control and cancer chemotherapy. J. Cell Biochem..

[28] Makin G., Dive C. (2001). Apoptosis and cancer chemotherapy. Trends Cell Biol..

[29] Furlan V., Konc J., Bren U. (2018). Inverse molecular docking as a novel approach to study anticarcinogenic and anti-neuroinflammatory effects of curcumin. Molecules.

