# Supplementary material for: Synthesis of MeON-Glycoside Derivatives of Oleanolic Acid by Neoglycosylation and Evaluation of Their Cytotoxicity against Selected Cancer Cell Lines
Source: Molecules. 2021 Feb 2;26(3):772. doi: 10.3390/molecules26030772 (PMC7867353; doi:10.3390/molecules26030772)
Supplement: Supplementary file 1 [file molecules-26-00772-s001.pdf]

Supporting information

Synthesis of MeON-Glycoside derivatives of oleanolic acid by neoglycosylation and evaluation of cytotoxicity against selected cancer cell lines

Zhichao Du [1,†], Guolong Li [2,†], Xiaoyang Zhou [1] and Jian Zhang [1,3,*]

SUPPORTING INFORMATION CONTENTS

I. ^{1}H NMR anomeric proton and HRMS characterization of C-3 or C-28 MeON-neoglycosides of oleanolic acid S2

Ⅱ. NMR spectra of neoaglycone and representative neoglycosides S4

I. **Table S1.** ^{1}H NMR anomeric proton and HRMS characterization of C-3 or C-28 MeON-neoglycosides of oleanolic acid

Entry	neoglycoside	*α*-anomeric H1		*β*-anomeric H1		*α: β*	HRMS(ESI)MS m/z	
		δ (ppm)	J(Hz)	δ (ppm)	J(Hz)	ratio	measured	calculated
**4a**	D-glucose			4.83-4.81[d]		1 anomer	706.4525[a]	706.4525
**4b**	L-glucose	not observed		4.84	8.7	Only *β*	706.4533[a]	706.4525
**4c**	2-deoxy-D-glucose			4.81-4.79 [d]		1 anomer	690.4575[a]	690.4576
**4d**	3-O-methyl-D-glucose			4.82-4.76 [d]		1 anomer	720.4678[a]	720.4681
**4e**	D-galactose	5.34	5.7	4.87-4.81[c]	n/d[e]	n/d	706.4520[a]	706.4525
**4f**	2-deoxy-D-galactose	not observed		4.76	10.9	Only *β*	690.4581[a]	690.4576
**4g**	D-mannose	4.93	1.7	4.70-4.67[c]	n/d	n/d	706.4528[a]	706.4525
**4h**	D-arabinose	5.33	5.4	not observed		Only *α*	676.4413[a]	676.4419
**4i**	L-arabinose	5.32	5.4	4.64	9.0	1:1	676.4411[a]	676.4419
**4j**	D-fucose	5.29	5.3	4.70	9.1	2:1	690.4585[a]	690.4576
**4k**	L-fucose	5.31	5.3	4.72	9.0	2:1	688.4434[b]	688.4430
**4l**	D-xylose	not observed		4.70	8.4	Only *β*	676.4426[a]	676.4419
**4m**	L-xylose	not observed		4.72	8.2	Only *β*	676.4430[a]	676.4419
**4n**	L-lyxose	not observed		5.18	8.2	Only *β*	676.4417[a]	676.4419
**4o**	L-rhamnose	4.67	2.0	5.14-5.09[c]	n/d	n/d	690.4578[a]	690.4576
**4p**	D-ribose	5.41	2.9	5.08	8.2	1:2	676.4423[a]	676.4419
**4q**	L-ribose	5.42	3.7	5.10	8.6	1:2	676.4413[a]	676.4419
**4r**	2-deoxy-D-ribose	5.32	2.9	4.66-4.62[c]	n/d	n/d	658.4326[b]	658.4324
**8a**	D-glucose	not observed		4.61	7.9	Only *β*	634.4676[a]	634.4677

**8b**	L-glucose	not observed		4.60	8.0	Only *β*	634.4673[a]	634.4677
**8c**	2-deoxy-D-glucose	not observed		4.72	10.5	Only *β*	618.4730[a]	618.4728
**8d**	3-O-methyl-D-glucose	not observed		4.63	8.9	Only *β*	648.4825[a]	648.4834
**8e**	D-galactose	4.67	2.8	5.11	6.2	4:1	634.4676[a]	634.4677
**8f**	2-deoxy-D-galactose	5.36	6.5	4.68-4.63[c]	n/d	n/d	618.4739[a]	618.4728
**8g**	D-mannose	5.00	2.5	4.77-4.70[c]	n/d	n/d	634.4672[a]	634.4677
**8h**	D-arabinose	5.18	5.5	4.67-4.65[c]	n/d	n/d	604.4581[a]	604.4572
**8i**	L-arabinose	5.18	5.9	4.63-4.59[c]	n/d	n/d	604.4576[a]	604.4572
**8j**	D-fucose			4.55-4.51[d]		1 anomer	618.4733[a]	618.4728
**8k**	L-fucose	not observed		4.44	8.7	Only *β*	618.4732[a]	618.4728
**8l**	D-xylose	not observed		4.57	6.7	Only *β*	604.4572[a]	604.4572
**8m**	L-xylose	not observed		4.55	8.7	Only *β*	604.4587[a]	604.4572
**8n**	L-lyxose	5.88	3.5	4.77-4.75[c]	n/d	n/d	604.4575[a]	604.4572
**8o**	L-rhamnose	4.69	2.9	5.28	6.1	4:1	618.4733[a]	618.4728
**8p**	D-ribose	5.23	3.4	4.80-4.77[c]	n/d	n/d	604.4580[a]	604.4572
**8q**	L-ribose	5.20	3.1	4.81-4.78[c]	n/d	n/d	604.4575[a]	604.4572
**8r**	2-deoxy-D-ribose	5.30	3.3	4.70-4.65[c]	n/d	n/d	588.4616[a]	588.4623
**1a**	D-glucose	not observed		6.34	8.0	Only *β*	641.4029[f]	641.4024
**1b**	D-glucose	not observed		4.92	7.7	Only *β*	627.4231[f]	627.4231

[a] HRMS (ESI) m/z for $[M+H]^+$, [b] HRMS (ESI) m/z for $[M-H]^-$, [c] Anomeric proton obscured by another peak, [d] Single anomeric proton signal detected but obscured by another peak, [e] Not determined, [f] HRMS (ESI) m/z for $[M+Na]^+$.

II. Figure S1 NMR spectra of neoaglycone and representative neoglycosides

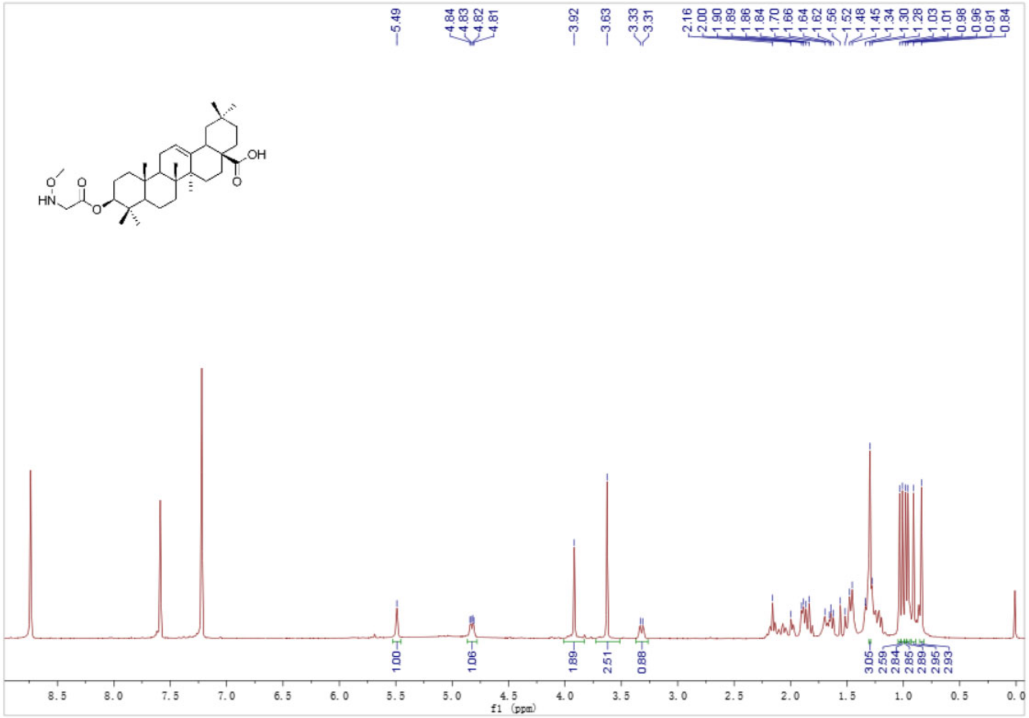

The ^{1}H NMR spectrum of compound **3** (500 MHz, in C_5D_5N)

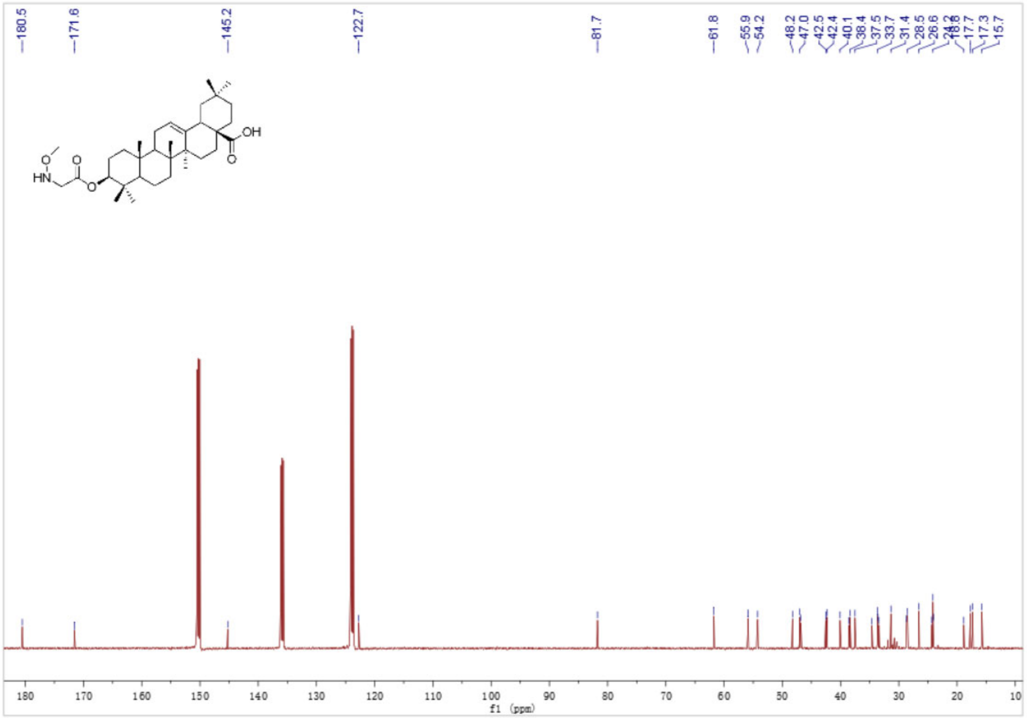

The ^{13}C NMR spectrum of compound **3** (125 MHz, in C_5D_5N)

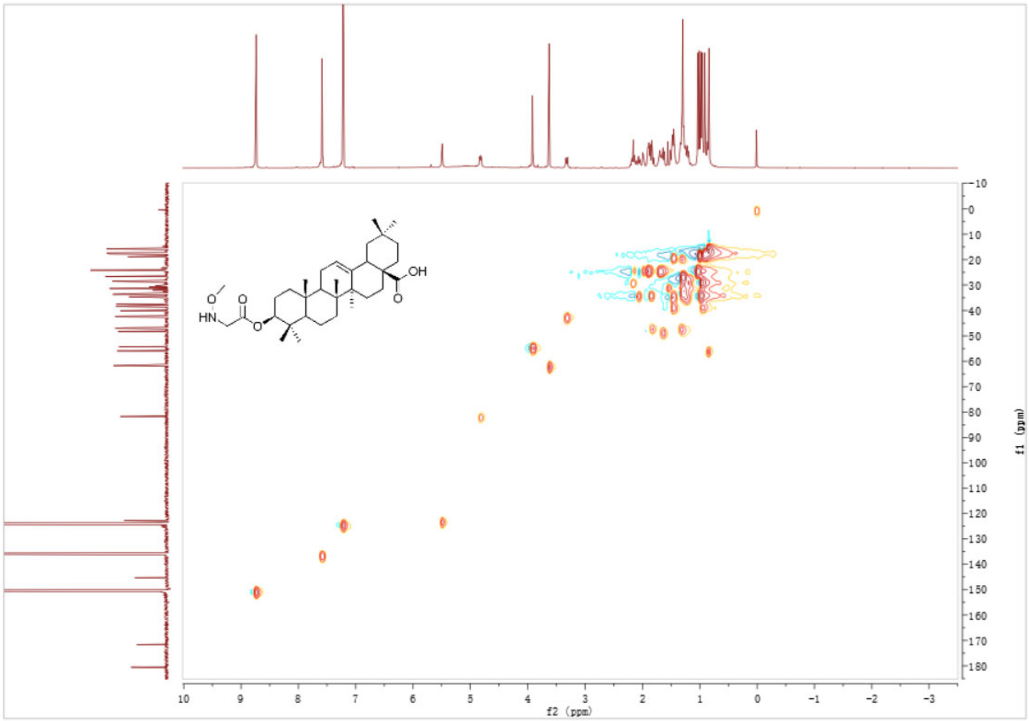

The HSQC spectrum of compound **3** (500 MHz, in C_5D_5N)

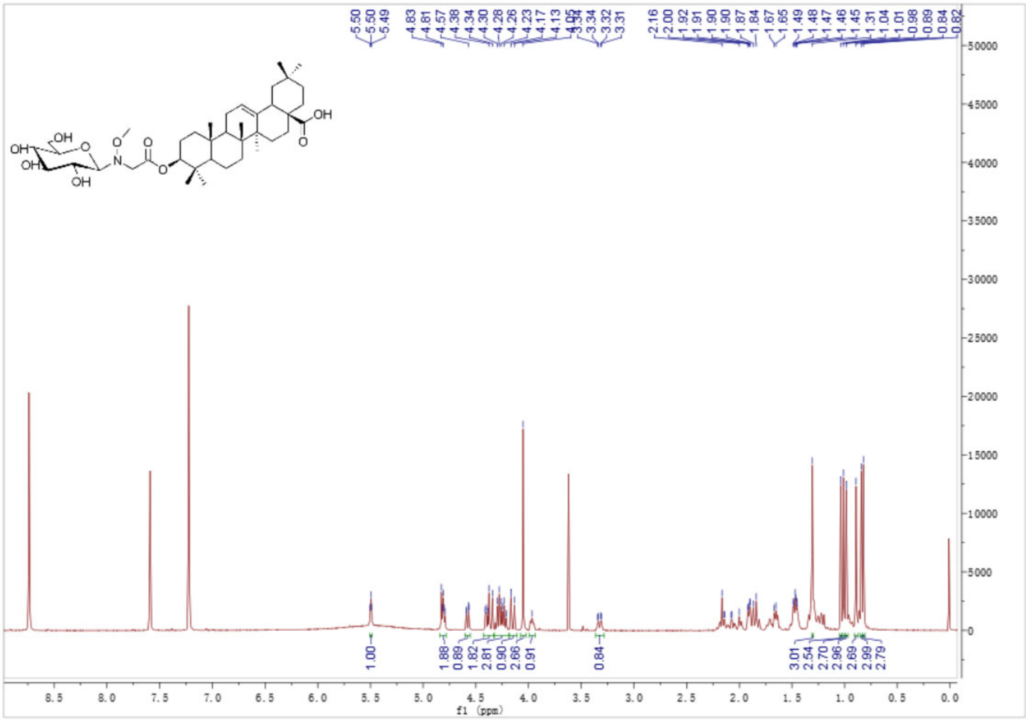

The ^{1}H NMR spectrum of compound **4a** (500 MHz, in C_5D_5N)

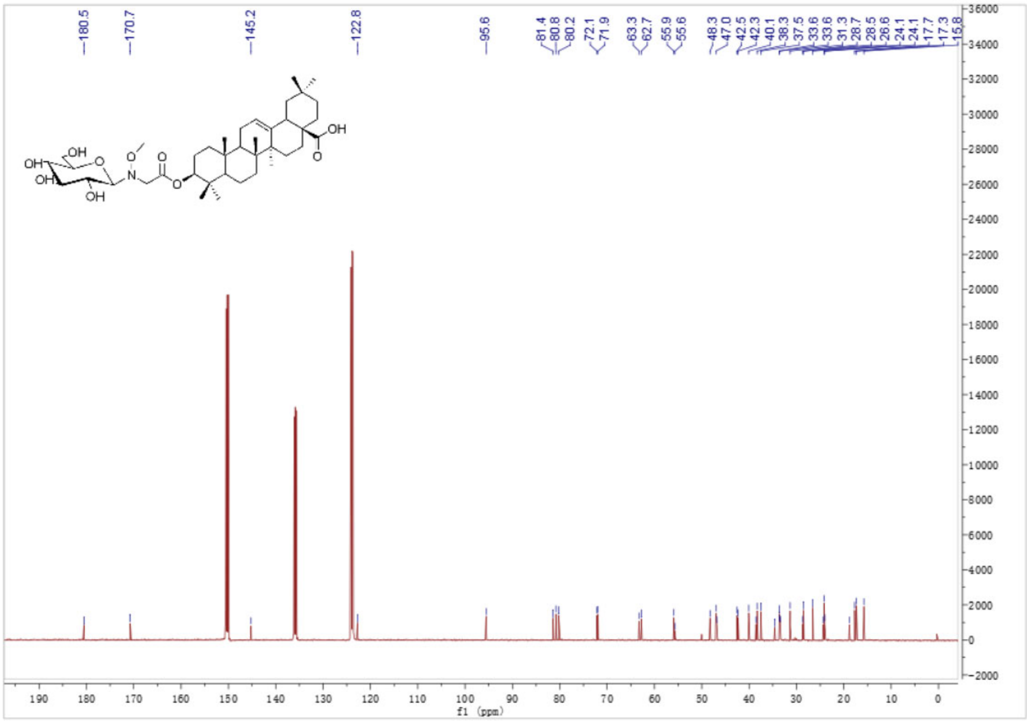

The ^{13}C NMR spectrum of compound **4a** (125 MHz, in C_5D_5N)

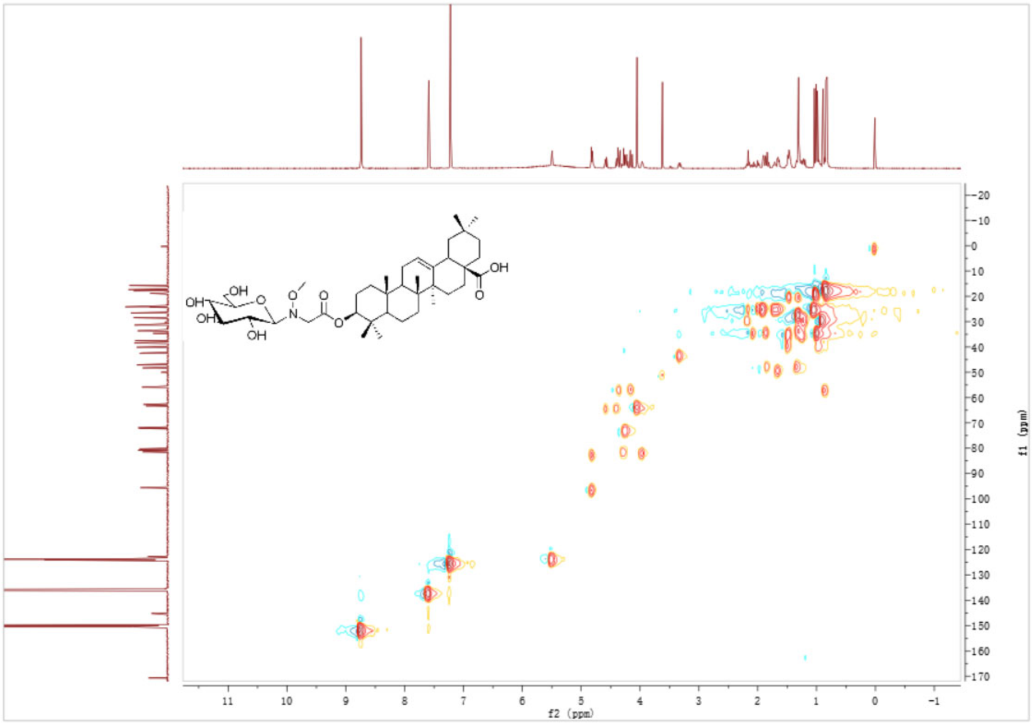

The HSQC spectrum of compound **4a** (500 MHz, in C_5D_5N)

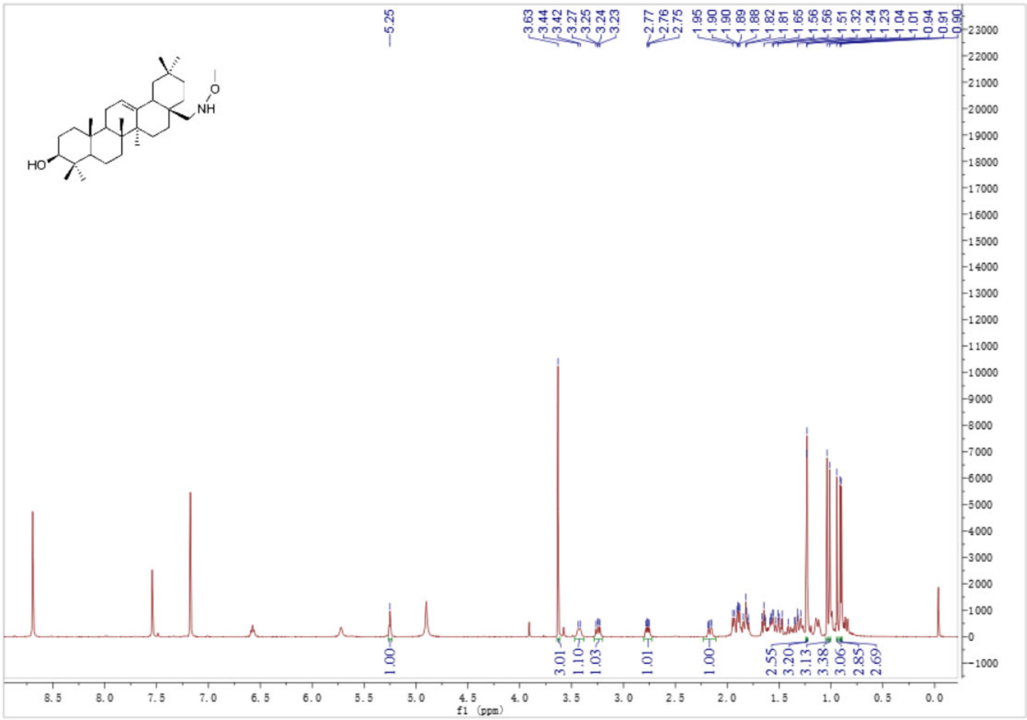

The ^{1}H NMR spectrum of compound **7** (500 MHz, in C_5D_5N)

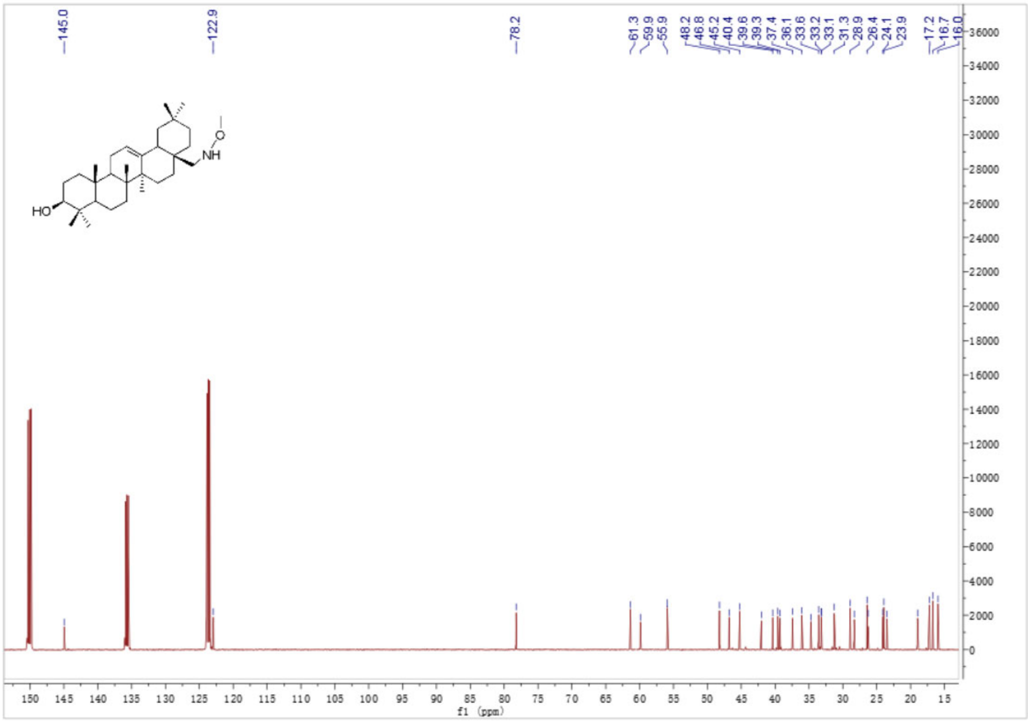

The ^{13}C NMR spectrum of compound **7** (125 MHz, in C_5D_5N)

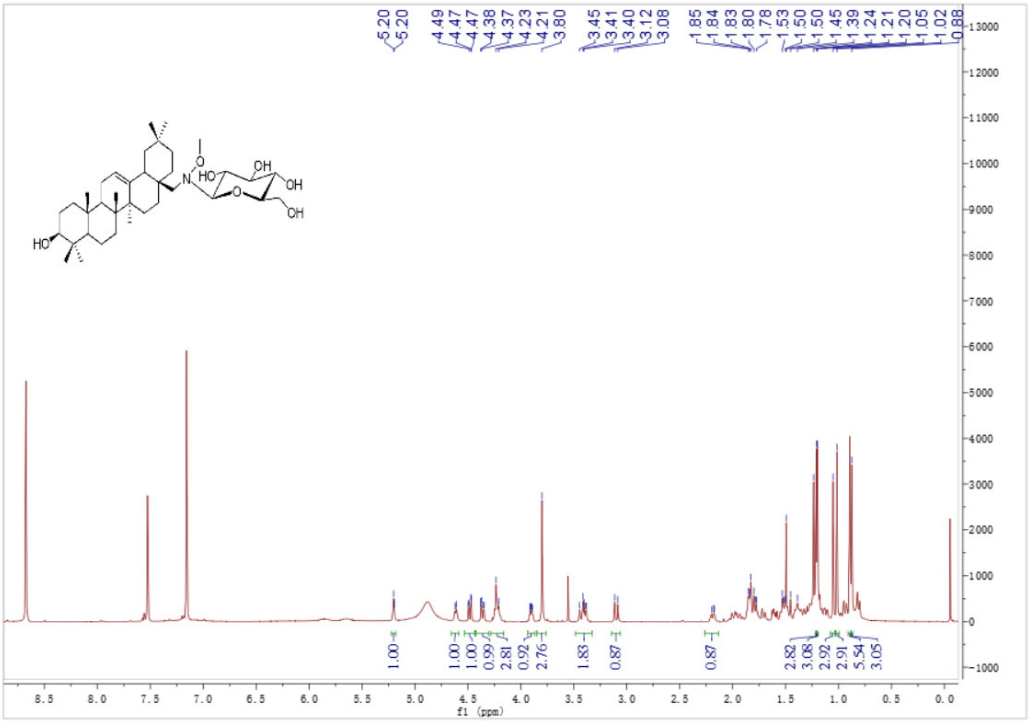

The ^{1}H NMR spectrum of compound **8a** (500 MHz, in C_5D_5N)

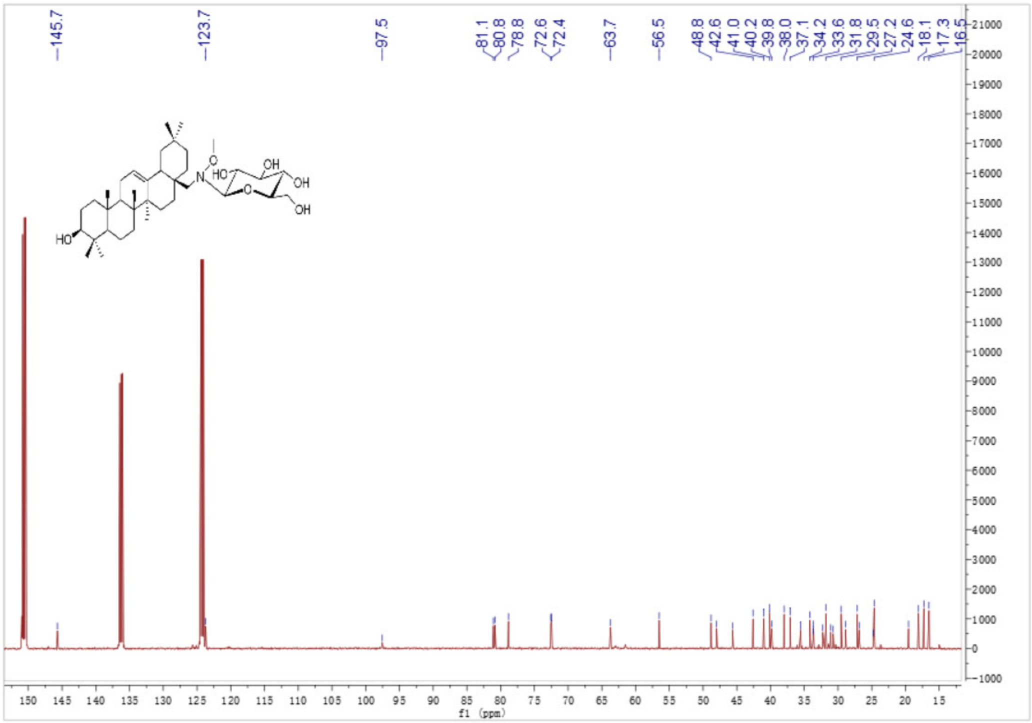

The ^{13}C NMR spectrum of compound **8a** (125 MHz, in C_5D_5N)

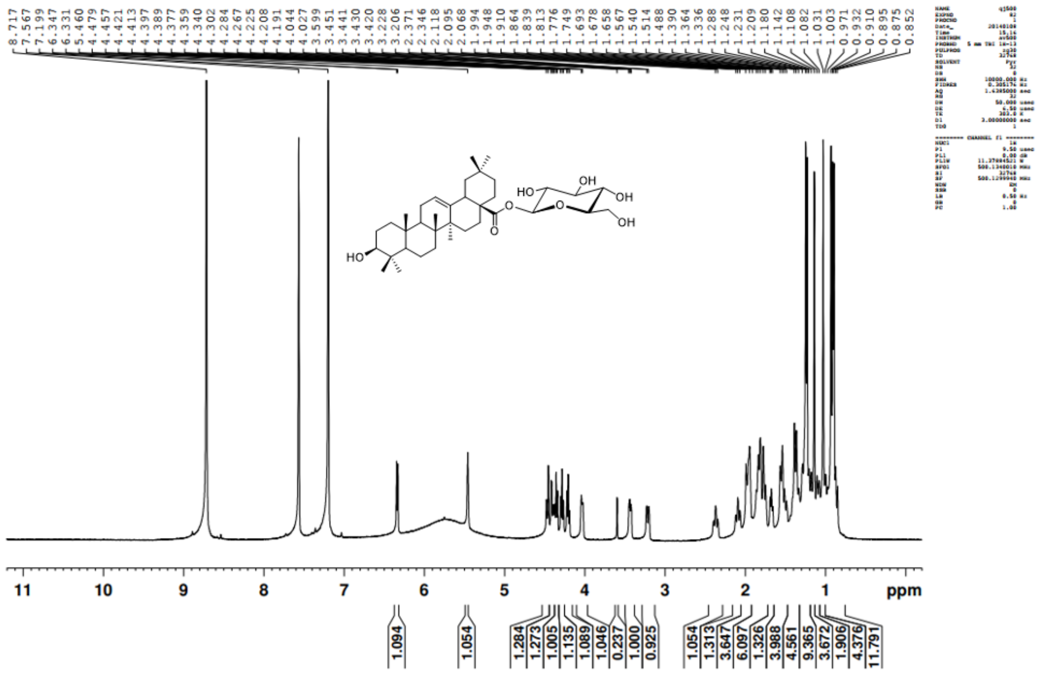

The ^{1}H NMR spectrum of compound **1a** (500 MHz, in C_5D_5N)

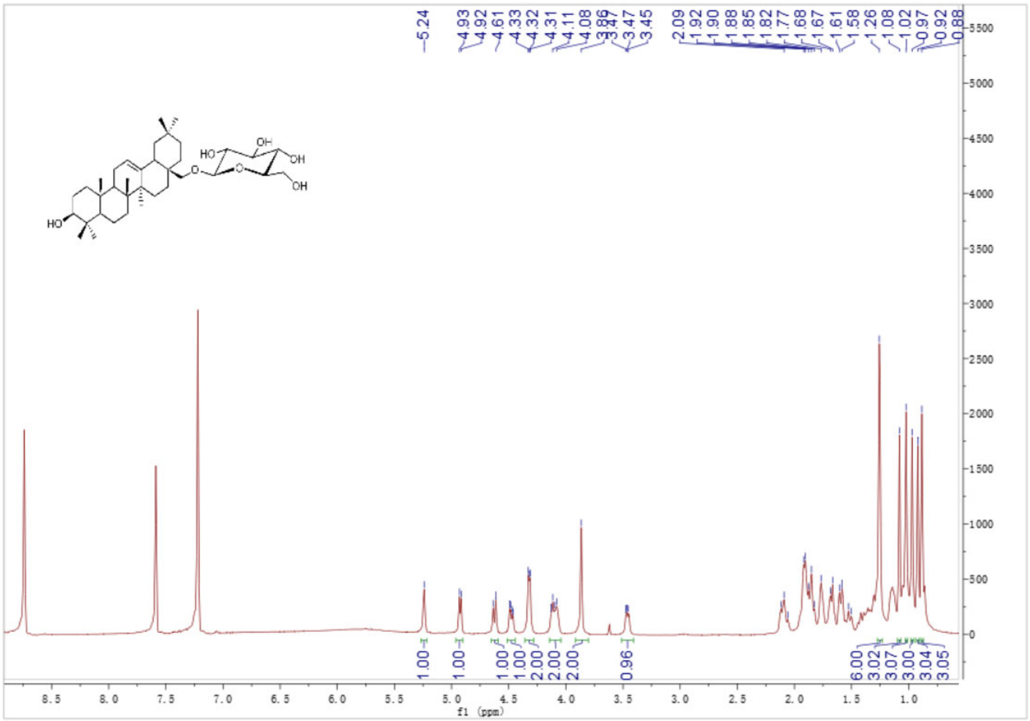

The ^{1}H NMR spectrum of compound **1b** (500 MHz, in C_5D_5N)
